# Lipidomics of Brain Tissues in Rats Fed Human Milk from Chinese Mothers or Commercial Infant Formula

**DOI:** 10.3390/metabo9110253

**Published:** 2019-10-28

**Authors:** Miya Su, Arvind K. Subbaraj, Karl Fraser, Xiaoyan Qi, Hongxin Jia, Wenliang Chen, Mariza Gomes Reis, Mike Agnew, Li Day, Nicole C. Roy, Wayne Young

**Affiliations:** 1 State Key Laboratory of Dairy Biotechnology, Dairy Research Institute, Bright Dairy and Food Co. Ltd., Shanghai 200436, China; shumiya@brightdairy.com (M.S.); qixiaoyan@brightdairy.com (X.Q.); jiahongxin@brightdairy.com (H.J.); chenwenliang@brightdairy.com (W.C.); 2 AgResearch Ltd., Grasslands Research Centre, Palmerston North 4442, New Zealand; Karl.Fraser@agresearch.co.nz (K.F.); Mariza.GomesReis@agresearch.co.nz (M.G.R.); Michael.Agnew@agresearch.co.nz (M.A.); Li.Day@agresearch.co.nz (L.D.); roynnz33@gmail.com (N.C.R.); Wayne.Young@agresearch.co.nz (W.Y.); 3 Riddet Institute, Massey University, Palmerston North 4474, New Zealand; 4 High-Value Nutrition National Science Challenge, Auckland 1023, New Zealand

**Keywords:** human milk, infant formula, lipids, composition, rat, brain, lipidomics

## Abstract

Holistic benefits of human milk to infants, particularly brain development and cognitive behavior, have stipulated that infant formula be tailored in composition like human milk. However, the composition of human milk, especially lipids, and their effects on brain development is complex and not fully elucidated. We evaluated brain lipidome profiles in weanling rats fed human milk or infant formula using non-targeted UHPLC-MS techniques. We also compared the lipid composition of human milk and infant formula using conventional GC-FID and HPLC-ELSD techniques. The sphingomyelin class of lipids was significantly higher in brains of rats fed human milk. Lipid species mainly comprising saturated or mono-unsaturated C18 fatty acids contributed significantly higher percentages to their respective classes in human milk compared to infant formula fed samples. In contrast, PUFAs contributed significantly higher percentages in brains of formula fed samples. Differences between human milk and formula lipids included minor fatty acids such as C8:0 and C12:0, which were higher in formula, and C16:1 and C18:1 n11, which were higher in human milk. Formula also contained higher levels of low- to medium-carbon triacylglycerols, whereas human milk had higher levels of high-carbon triacylglycerols. All phospholipid classes, and ceramides, were higher in formula. We show that brain lipid composition differs in weanling rats fed human milk or infant formula, but dietary lipid compositions do not necessarily manifest in the brain lipidome.

## 1. Introduction

The general consensus is that human milk is the preferred source of nutrition for the infant [1,2]. Designing infant formulae to mimic the composition of human milk is therefore common practice [3]. Several studies focusing on cognitive and associated neurodevelopment of infants favor breast feeding compared to formula-based diets [4,5,6]. Although socioeconomic status, genetics, and environmental predisposition are partly implicated for the results in favor of human milk [4,6], differences in the nutritional composition of human milk and formula can have a huge impact on mental development [6], particularly white matter [5]. Key components found at higher concentration in human milk include polyunsaturated fatty acids, cholines, and phospholipids [6,7]. Although these compounds are normally added to infant formula, the ingredients are mostly derived from plant sources, which have different molecular structures compared to those in human milk. With other natural health benefits of human milk [8,9] such as immunity, infant formula product manufacturers are constantly aiming for improved formula composition and function towards human milk. Nevertheless, there are times where breast feeding the infant may not be possible, mainly where the composition of human milk may be altered due to environmental, pharmacological, or physiological factors. Under such circumstances, alternative milk-based nutrition is the only choice [1]. The holistic growth and development of the infant is critical, and lipids are an important component of milk in terms of both nutrition and the physical characteristics that they impart. 

Lipids function as an energy supply for muscles [10], heart, and liver, and are incorporated into cell membranes and nerve tissue for the development and maintenance of the body function. They are also essential for the synthesis of molecules in the human body via enzymatic processes (including the synthesis of steroid hormones), in the transmission of membrane signals, and for the transport of fat-soluble vitamins [2]. Lipids are present in the form of globules enclosed in milk fat globular membrane (MFGM) structures [11], representing the major source of energy and nutrients for infants that are required for normal development. Triacylglycerols constitute 96–98% of milk fat, along with other neutral lipids (e.g., free fatty acids and cholesterol), polar lipids (e.g., phospholipids), and lipo-soluble compounds (e.g., fat-soluble vitamins) [1]. Lipids are altered in the formula manufacturing process, and therefore, in an attempt to match the composition of human milk, plant sourced lipids are often added to infant formula. These lipid ingredients lack or are low in certain lipid classes that are naturally present in human milk, such as long chain-polyunsaturated fatty acids (LC-PUFAs) or their precursors [12], MFGMs [13], and phospholipids (PLs) [14].

In spite of fortification of infant formula with key ingredients that enhance cognitive behavior, human milk is still considered the best resource for cognitive development and I.Q. [1]. This is mainly attributed to other unknown resources such as body fat stores which may play a pivotal role in lipid availability [1], and the lack of a full understanding of differential digestibility and availability of lipids between human milk and infant formula [10]. Lipidomics, the analysis and characterization of lipid classes and species in cells, tissues, and biofluids [15], provides a comprehensive coverage of lipids in biological samples. A comparison of the lipid profiles of rat brain samples fed human milk or infant formula, and simultaneously the lipid profiles of human milk and formula samples themselves, will reflect upon the differential bioavailability of these dietary lipids as well as other precursor metabolites within the milks.

In a previous study, we showed that human milk and infant formula differentially modified the gut tissue transcriptome and proteome, and the plasma metabolome in a growing rat model [16]. Here in this new study, we compared lipid compositions of human milk and infant formula and conducted a non-targeted lipidomics analysis of the whole brain of rats from the previous study to better understand the impact of dietary lipids on the brain lipidome.

## 2. Results

### 2.1. Rat Brain Lipidomics

#### 2.1.1. Lipid Annotation and Identification

Criteria for lipid annotation and identification were described in materials and methods. Here, the manual identification of PE(16:0/18:1) + H, fulfilling the identification criteria and displaying a match with LipidSearch^TM^ results, is shown in Figure S1. EIC of the parent ion *m*/*z* 718.5392 at 387.28 secs (6.45 min) is shown in Figure S1A. Elemental composition of the parent ion matching with the molecular weight of C_39_H_77_O_8_NP for PE(16:0/18:1) + H (±1.45 ppm) is shown in Figure S1B. Product ions of *m*/*z* 718.54 (CE = 30 eV) based on ddMS^2^ is shown in Figure S1C, and principal product ions corresponding to an analytical standard of PE(16:0/18:1) in the LipidMaps database is shown in Figure S1D. Figure S1E shows the mass spectrum of theoretical product ions for PE(16:0/18:1) + H from LipidSearch^TM^, matching with the spectrum of the analytical standard (Figure S1D) and raw file (Figure S1C). A list of tentative product ions and their formulae is provided in Table S1. *m*/*z* 577 corresponds to a neutral loss of *m*/*z* 141, diagnostic of PE [17]. Finally, Table S2 shows a list of putative combinations of fatty acids corresponding to PE(34:1), with the 16:0/18:1 and 18:1/16:0 combinations scoring highest with a m-score of 29.8.

A total of 163 lipid species, 110 in positive and 53 in negative ionization modes, were identified by LipidSearch and matched with XCMS results (Table S3).

#### 2.1.2. Lipid Composition of Rat Brain Samples (Human Milk vs. Infant Formula)

**Figure 1:**
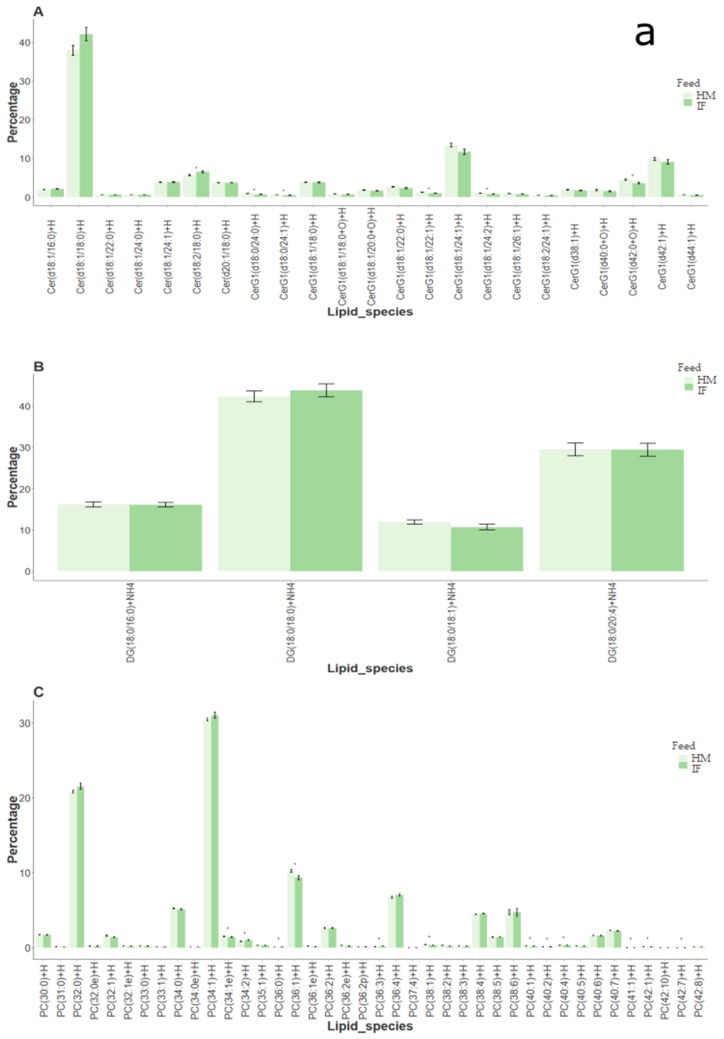

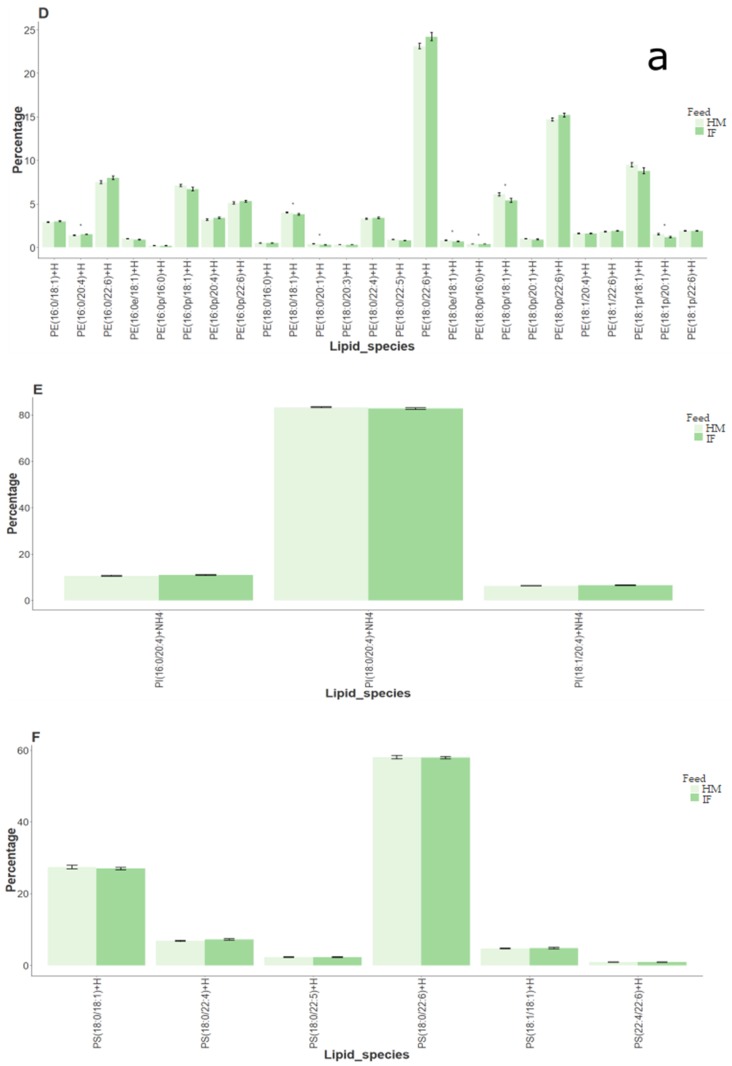

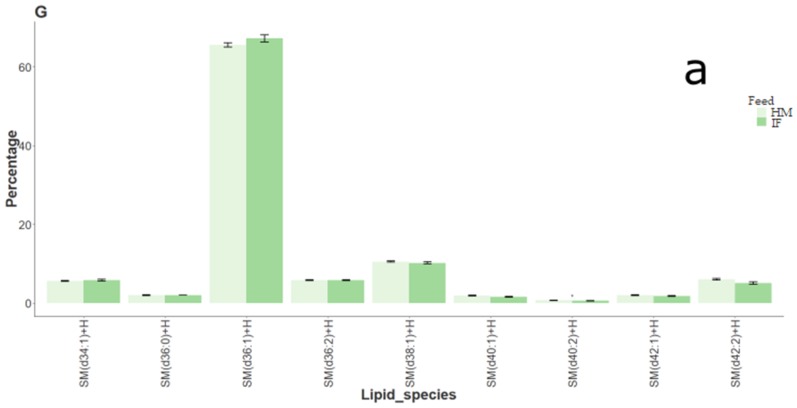

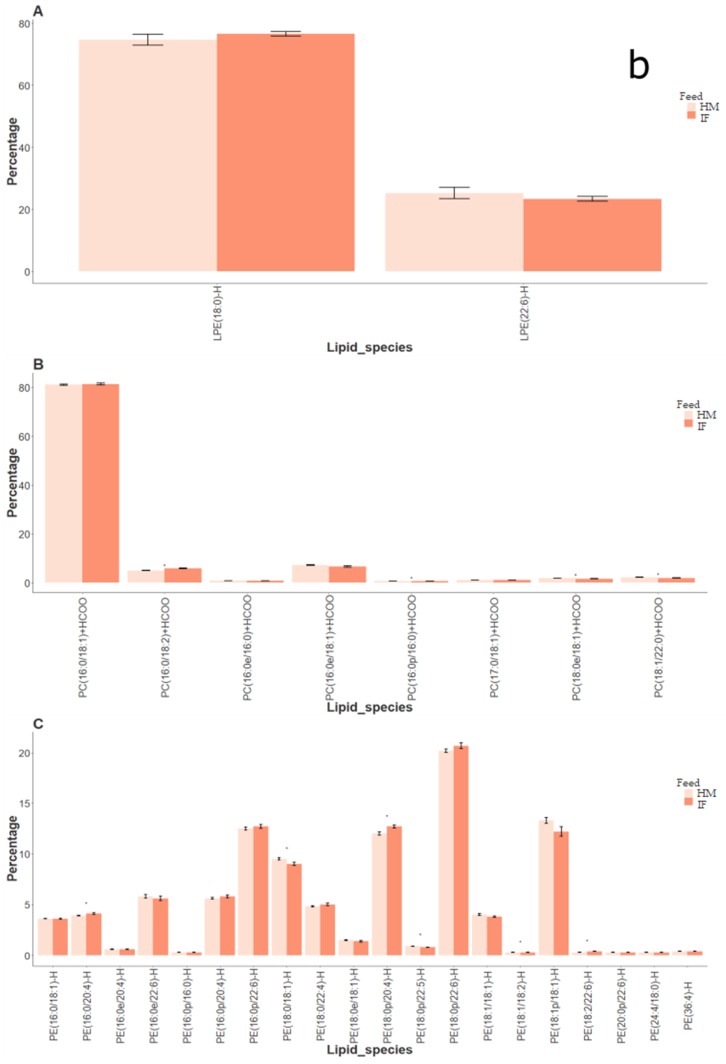

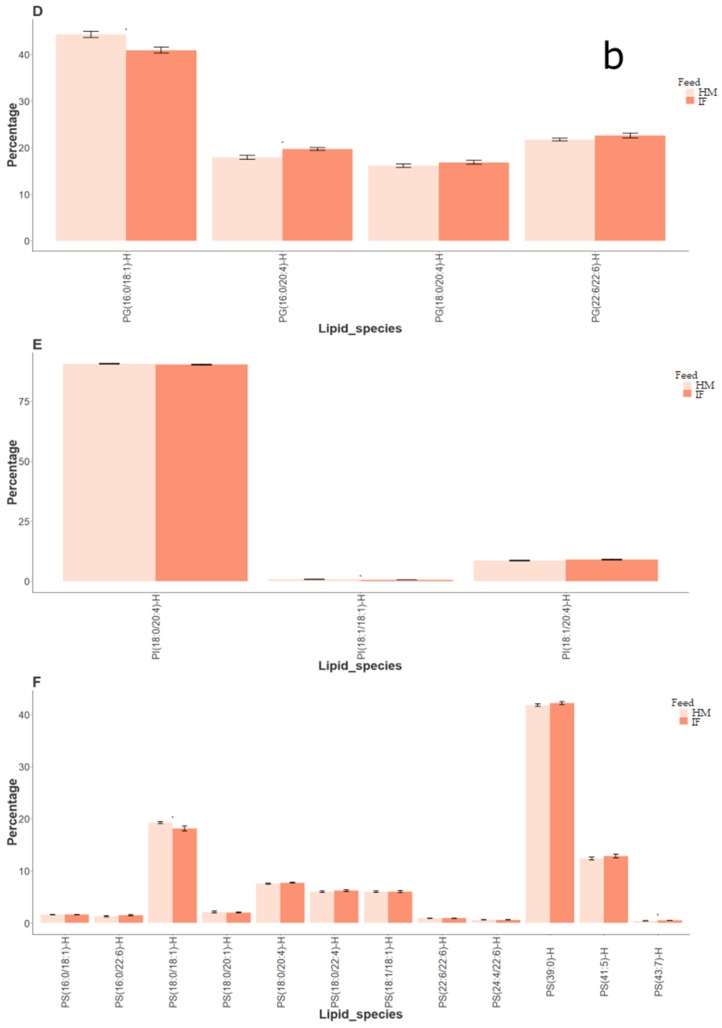

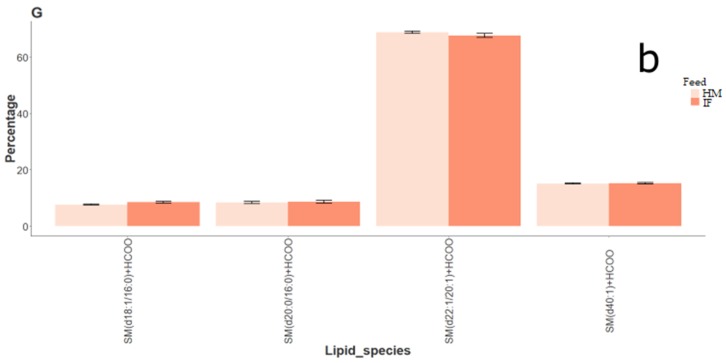
Class and species composition (percentage) of lipids in the brains of rats fed human milk or infant formula (*n* = 12), in (**a**) positive and (**b**) negative ionization modes of lipidomics analysis. In positive mode: (**A**) Cer—Ceramides; (**B**) DG—Diacylglycerol; (**C**) PC—Phosphatidylcholine; (**D**) PE—Phosphatidylethanolamine; (**E**) PI—Phosphatidylinositol; (**F**) PS—Phosphatidylserine; and (**G**) SM—Sphingomyelins. In negative mode: (**A**) LPE—Lysophosphatidylethanolamine; (**B**) PC—Phosphatidylcholine; (**C**) PE—Phosphatidylethanolamine; (**D**) PG—Phosphatidylglycerol; (**E**) PI—Phosphatidylinositol; (**F**) PS—Phosphatidylserine; and (**G**) SM—Sphingomyelins.

**Figure 2:**
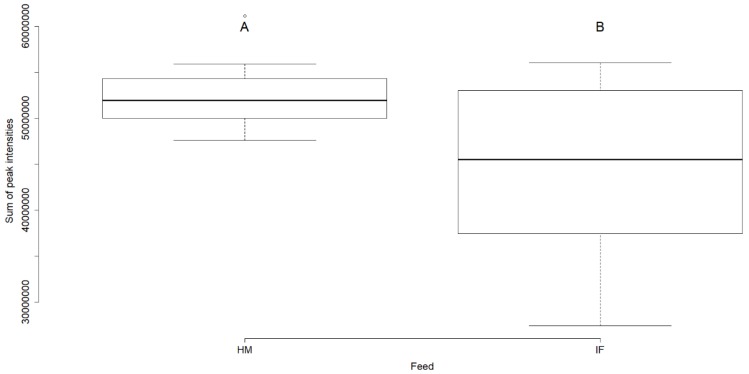
Boxplot of the overall brain sphingomyelin (SM) concentrations in negative ionization mode of rats fed human milk or infant formula. Mean separation by Fisher’s LSD (different upper case letters indicate significant difference at α = 0.05; *n* = 12).

Peak intensities of lipid species were summed into their respective classes for each ionization mode separately, and the percentage contribution of each species respective to its class in both human milk and formula fed rat brain lipidomes is shown in Figure 1. A total of seven lipid classes were present in each ionization mode (Figure 1). Of the lipid classes identified in positive and negative ionization modes, only the SM class was found in significantly higher concentrations in the brains of rats fed human milk (α = 0.05; Figure 2).

Lipid species that were significantly different (α = 0.05) in percentage contributions to their respective classes are shown in Table S4. In positive ionization mode, 6 ceramide (out of 23), 12 PC (out of 40), 7 PE (out of 25), and 1 SM (out of 9) species—i.e., a total of 26 species out of 110—were significantly different between human milk and infant formula fed samples, of which, 22 were higher in human milk samples (Figure 1a; Table S4). Species high in human milk fed samples predominantly contained C18:0 or C18:1 fatty acid, although all PCs were presented as the sum of their fatty acids (Table S4). Cer(d18:1/18:0) + H, DG(18:0/18:0) + NH_4_, PC(34:1) + H, PE(18:0/22:6) + H, PI(18:0/20:4) + NH_4_, PS(18:0/22:6) + H, and SM(d36:1) + H contributed the highest percentage in their respective classes (Figure 1a).

In negative ionization mode, 4 PC (out of 8), 6 PE (out of 20), 2 PG (out of 4), 1 PI (out of 3), and 2 PS (out of 12) species—i.e., a total of 15 species out of 53—were significantly different between human milk and infant formula fed samples, of which, 8 were higher in human milk samples (Figure 1b; Table S4). Again, the species high in human milk fed samples predominantly contained C18:0 or C18:1 fatty acid, while species high in infant formula samples predominantly contained 20:4 or 22:6 fatty acid (Table S4). LPE(18:0) − H, PC(16:0/18:1) + HCOO, PE(18:0p/22:6) − H, PG(16:0/18:1) − H, PI(18:0/20:4) − H, PS(39:0) − H, and SM(d22:1/20:1) + HCOO were the highest in their respective classes (Figure 1b). Some species such as PC(34:1), PE(18:0/22:6), and PI(18:0/20:4) were common between positive and negative ionisation modes. 

### 2.2. Human Milk and Infant Formula Composition

#### 2.2.1. Fatty Acid Composition

A diverse range of fatty acids (FAs), from C4:0 to C24:1, were found in human milk and infant formula samples (Table 1). Palmitic (C16:0), oleic (C18:1) and linoleic acids (C18:2) were predominant in both samples. Minor FAs such as C8:0 and C12:0 were high in formula, whereas C16:1 and C18:1 n11 were higher in the human milk samples (Table 1).

#### 2.2.2. Triacylglycerol (TAG) Composition

Considerable differences were observed in overall distributions of TAG composition between human milk and infant formula samples (Table 2). Generally, formula samples had higher levels of the low- to medium-carbon TAGs, while human milk samples had higher levels of the high-carbon TAGs (Table 2). The exact FA compositions of these TAGs, and their positions in the glycerol backbone were not determined.

#### 2.2.3. Phospholipid Composition

A comparison of the distribution and absolute concentrations (mg/100 mL) of PLs between human milk and infant formula samples, showed all PL classes (PI, PE, PS, PC, and SM) were higher in formula (Table 3). 

## 3. Discussion

### 3.1. Rat Brain Lipidomics

Fats are the major source of energy for the growing infant, and their composition (FAs, TAGs, and PLs), as observed in the current study (Table 1, Table 2 and Table 3, respectively), differs significantly between human milk derived from animal fats, and infant formulae mainly derived from plant fats [3,14]. In spite of differences in composition of human milk and infant formula, significant differences in the lipid composition of brains was not observed. Of all the lipid classes identified in rat brains fed human milk or infant formula, only SM was found at higher concentrations in brains of human milk fed samples (Figure 2). Notably, SM concentrations were higher in infant formula (Table 3). Lipid species that contributed highest concentrations in their respective classes in both positive (Figure 1a) and negative (Figure 1b) ionization modes, were similar in both human milk and infant formula fed brain samples, further corroborating the limited impact of sample compositions on the brain lipidome. Though diets are a significant source of lipids that determine cognitive behavior [1], stable isotope studies have shown that body fat stores are also key determinants of lipid availability, and are able to metabolically buffer short-term changes in the dietary lipid composition [2]. 

Fat digestion and availability occurs through several phases: salivary, gastric and intestinal, ultimately resulting in the release of glycerol and free-FAs [10]. This process, among other factors in human milk is determined by MFGMs, which control FA and TAG composition [11], and in formula by the stereospecific position of FAs [10]. In fact, infant formula supplemented with MFGM has proven beneficial effects on cognitive behavior [13]. This again reiterates the significance of tailoring infant formula to mimic human milk, where MFGMs are known to contain significant amounts of PC and SM [18], and thereby promote cognition. Differences in intestinal microbiota of rats fed human milk or infant formula have been observed in a similar study [16]. This in turn may have an indirect role in modifying the digestion and absorption of lipids [19] observed in the current study. However, caution is advised in extrapolating these results to human physiology because significant differences in fecal microbiota was not observed in babies fed the same human milk or infant formula [16]. Clearly, a combination of compositional differences in lipids between human milk and infant formula, differences in the digestion and absorption of fats, and host physiology determine bioavailability of lipids.

PC, PE, PI, PS, PG, DG, LPE, Cer, and SM were the major lipid classes identified in brain samples (Figure 1). These results are in accordance with published literature on the lipid composition of brain samples, where glycerophospholipids comprising PC, PE, PS, and PI, and sphingolipids comprising SM and Cer are predominant in the brain plasma membrane [20]. While major differences in the rat brain lipidome based on human milk or infant formula diets were not evident (Figure 1), SM was significantly higher in rat brains fed human milk (Figure 2). Choline, the precursor to the biosynthesis of PLs PC and SM, which in turn are precursors for Cer [7], is capable of circulation and is crucial for brain function [18]. Digestion and uptake of the choline-containing classes (PC, SM) are regulated by several factors, where PC is digested independent of choline, SM is absorbed in the intact form, and choline is absorbed from the duodenum, jejunum, and ileum [18]. A possible explanation for the significantly high concentrations of the lipid species of these choline-containing classes in rat brain samples fed human milk, is the differential breakdown of these classes in the intestine, and henceforth transport and accumulation of choline in the brain [18]. 

Lipid species that contributed significantly higher percentages in brain samples fed human milk compared to infant formula predominantly contained C18:0 or C18:1 fatty acid (Figure 1; Table S4). Fatty acids of the brain have been reported to be consistently high in stearic acid (C18:0), with the *sn*-2 position primarily carrying mono- or polyunsaturated fatty acids [21], which fits with the suggested lipid structures in the current results (Table S4). Rosenberg and Stern [21] also observed changes in FA profiles and concentrations in rat brains, concomitant with the age of rats. C18 FAs remained the major FA group throughout all ages of development. However, there was an overall decline in stearic acid (C18:0) content with increasing age, and a concomitant increase in C18:1 FAs [21]. While the rats used in this study were of post-weaning age and used as a model for weaning, it is possible effects may be even larger if neonatal animals were used.

While PUFAs are well-known for their neuroprotective functions in the brain [22], the exact fatty acid compositions of the PCs denoted by a sum of the total carbons and double bonds—i.e., PC (40:4), (42:7) etc.—could not be easily deduced (Table S4). Lipid species containing arachidonic acid (AA; C20:4 n6) and docosahexaenoic acid (DHA; C22:6 n3), the LC-PUFAs mainly implicated in cognitive behavior, contributed significantly higher percentages in brain samples fed infant formula compared to human milk (Figure 1; Table S4). Both these fatty acids were at relatively similar concentrations in human milk and infant formula (Table 1). It was recently shown that fortifying infant formula with both DHA and AA, mimicking the composition of human milk, generated rat brain activity comparable to that of animals fed mother’s milk [23]. 

Nonetheless, we have shown that feeding human milk or infant formula in a rat model can lead to overall differences in total brain SM concentrations, although, spatial changes in the accumulation of FAs in different regions of the brain have been observed, depending on the amount of grey or white matter [12].

### 3.2. Human Milk and Infant Formula Lipid Composition

Essential FAs, linoleic acid (LA; C18:2 n6) and α-linolenic acid (ALA; C18:3 n3), were respectively found at higher proportions in human milk samples, or at equal proportions between human milk and infant formula samples (Table 1). These FAs are precursors to LC-PUFAs such as AA and DHA, which in turn are deposited in the growing brain, and are functionally related to cognitive development [2]. Though significant differences in the concentrations of AA and DHA between human milk and infant formula samples were not found (Table 1), conversion of ALA to DHA is possible, albeit at a lower rate (< 1%) in humans [24].

Though the proportion of palmitic acid (C16:0) was not significantly different between human milk and infant formula samples (Table 1), bioavailability is determined by its location in the triglyceride molecule [10]. A major portion of human milk palmitic acid is present in the *sn*-2 position, which is excluded from lipolytic enzyme action, thereby retaining palmitic acid as a monoglyceride which due its high water-solubility is readily available compared to free palmitic acid [2]. Again, in accordance with previous studies [14], oleic acid (C18:1 n9) was found at higher proportion in infant formula (Table 1). Although MUFAs can be synthesized by the body, diets enriched in oleic acid have shown to have some health benefits [2].

About 98% of total lipids in human milk are triacylglycerols (TAGs), whose characteristics and bioavailability are determined by the esterified FAs [14]. As observed in the current study (Table 2), medium-chain TAGs, mostly comprising FAs with 6–12 carbon atoms such as caproic, caprylic, or capric acids were also found at higher concentrations in infant formula in other studies, and rarely detected in human milk samples [14]. Addition of medium-chain TAGs in formulae facilitates better absorption of lipids in the gastrointestinal tract and have beneficial effects in children with intestinal malfunctions. However, they also supposedly limit the oxidation of PUFAs [3], which have higher significance in cognitive function. On the other hand, human milk samples had long-chain TAGs (Table 2) which is presumably related to high levels of LC-PUFAs [1].

Total PL, and all major PL classes, were found at higher absolute concentrations in infant formula (Table 3). Other studies have shown identical results [25], or higher concentrations of PC and SM in human milk compared to infant formula samples [7]. Among all PL classes, PC and SM are a significant source of choline to the infant [25]. Choline is a neurotransmitter, critical for brain development in the neonate, and is ingested mainly in the form of PC and SM, rather than the free base [7,18]. PLs are also essential components of the cell membrane of milk fat globule structures that encompass TAGs, and thereby modulate digestion and availability of LC-PUFAs, especially AA and DHA [14,26].

Human milk is a complex structure whose composition, and thereby function, are not fully understood [1]. This limits our understanding of differences in digestion, uptake, and availability of lipids from the diet to the brain lipidome. The increased levels of SM and linoleic acid (LA; C18:2 n6) in infant formula (Table 3) and human milk (Table 1) samples, respectively, did not translate to a corresponding increase of these classes or their biosynthetic products (AA and DHA) in the rat brain lipidome. Possible explanations are differences in digestion and therefore bioavailability of different constituents, innate fat stores, stereospecific positioning of FAs, and henceforth their susceptibility for degradation, amongst other factors. Further studies, especially stable isotope experiments, combined with spatial tracking of dietary lipids to different regions of the brain are required to fully decipher the impact of dietary lipids on the brain lipidome.

## 4. Materials and Methods

### 4.1. Sample Collection

Details of human milk collection and infant formula composition are as previously described [16]. Briefly, human milk samples were collected from 900 volunteer Chinese mothers, pooled, freeze-dried and stored at −20 °C. Prior to rat feeding or compositional analysis, samples (13 g) were thawed, and reconstituted in 100 mL of water. Synlait Pure Canterbury Formula was used for the infant formula treatment and reconstituted as per the recommended guidelines (15 g/100 mL of water). Nutritional composition analyses of both samples are provided in Table S5.

### 4.2. Rat Growth Conditions, Treatment, and Ethics

Male Sprague Dawley rats were weaned and caged individually at 21 days of age. Rats were randomly allocated to either human milk or infant formula fed groups (*n* = 12). Both samples were prepared fresh twice a day and fed to rats ad libitum for 28 days instead of drinking water. A modified AIN-93M adult rodent maintenance diet containing beef protein and soybean oil was also provided to all rats [27]. Other monitoring parameters are as described in Liu, Roy [16]. At the end of 28 days, rats were euthanized by CO_2_ overdose (AgResearch Animal Ethics Committee Approval number: AEC13099), and brain samples collected for lipidomics analyses. Samples were stored at −80 °C until sample preparation.

### 4.3. Rat Brain Lipidomics

Brain samples were thawed overnight (4 °C), homogenized in a bead-mill homogenizer (2 min @ 30 Hz; TissueLyser II; Qiagen, USA), and weighed (50 ± 5 mg) into a 2 mL micro-centrifuge tube. An extraction solvent comprising 800 µL of chloroform:methanol (1:1; *v*/*v*) was added, samples were homogenized again (2 min @ 30 Hz), diluted with water (400 µL), vortexed (30 s), and centrifuged (4 °C, 18,188 × *g*) for 10 min. The lower, organic layer was taken (200 µL) separately, evaporated to dryness under a continuous stream of nitrogen (30 °C), and the dried extract was reconstituted in 400 µL of chloroform:methanol (2:1; *v*/*v*), with 10 μg/mL of 16:0 d_31_-18:1 phosphatidylethanolamine as internal standard. Finally, samples were vortexed (60 s), and 100 μL was transferred to a glass insert in an auto-sampler vial for LC-MS analysis.

Powdered infant formula and human milk samples were also extracted for lipidomics. One (1) mL of chloroform:methanol (2:1 *v*/*v*, with 10 μg/mL of internal standard 16:0 d_31_-18:1 phosphatidylethanolamine) was added to 300 μL of milk, samples were vortexed (1 min), 300 μL of water was added, and centrifuged (4 °C, 18,188 × *g*) for 10 min, and 100 μL of the lower, organic layer was transferred to a glass insert in an auto-sampler vial for LC-MS analysis.

### 4.4. UHPLC-MS Conditions

Lipid analysis was performed using a Thermo UHPLC–MS system (Thermo Fisher Scientific, Waltham, MA, USA) comprising an Accela 1250 quaternary UHPLC pump, a PAL auto-sampler fitted with a 15,000 psi injection valve (CTC Analytics AG., Zwingen, Switzerland), a 2 μL injection loop, and a Q-Exactive Orbitrap^TM^ mass spectrometer with electrospray ionization. Samples were cooled in the auto-sampler at 4 °C and 2 µL of extract was injected into a Waters Acquity CSH-C18 column (100 × 2.1 mm ID; 1.7 µm particle size) at 65 °C and eluted over a 17 min gradient with a flow rate of 600 μL/min. The mobile phase was a mixture of isopropanol:acetonitrile (90:10, *v*/*v* with 0.1% formic acid and 10 mM ammonium formate) (solvent A) and acetonitrile:water (60:40, *v*/*v* with 0.1% formic acid and 10 mM ammonium formate) (solvent B). The gradient elution program was as follows: 15–30% A (0–2 min), 30–48% A (2–2.5 min), 48–82% A (2.5–11 min), 82–99% A (11–11.5 min), held at 99% A (11.5–14 min), 99–15% A (14–14.1 min), held at 15% A (14.1–17 min).

Both full and data dependent MS^2^ (ddMS^2^) scans were collected in profile data acquisition mode. For full scan mode, a mass resolution setting of 35,000 was set to record a mass range of *m*/*z* 200–2000 with a maximum trap fill time of 250 ms. In ddMS^2^, MS^2^ measurements are activated when a set peak intensity threshold is achieved. For ddMS^2^ scan mode, the same mass resolution setting was maintained with a maximum trap fill time of 120 ms. The isolation window of selected MS^1^ scans was ± 1.5 *m/z* with a normalized collision energy of 30. Samples were run in both positive and negative ionization modes separately. Positive ion mode parameters were as follows: spray voltage, 4.0 kV; capillary temperature, 275 °C; capillary voltage, 90 V; tube lens 120 V. Negative ion mode parameters were as follows: spray voltage, −2.5 kV; capillary temperature, 275 °C; capillary voltage, −90 V; tube lens, −100 V. The nitrogen source gas desolvation settings were the same for both modes (arbitrary units): sheath gas, 40; auxiliary gas, 10; sweep gas, 5. The Xcalibur software package provided by the manufacturer was used to create these settings.

#### 4.4.1. Run Sequence

The sequence of runs comprised blanks, QCs and samples in that order. The QC comprised a pooled aliquot of the extract of all samples. Each sample was analyzed in positive and negative ionization modes in sequential order. 

To verify and maintain data quality, the QC sample was injected once every 10 samples. Retention time, signal intensity, and mass error of the internal standard were constantly monitored during the runs. For every 10 samples, one sample was selected at random for data dependent MS^2^ (ddMS^2^). Fragmentation data on approximately 4 samples in total per ionization mode (positive and negative) were used for identification of lipid ions/classes.

#### 4.4.2. Data Analysis

Raw data files (Thermo .raw files), consisting of blanks, QC, and study samples were converted to mzXML files using MSConvert function of ProteoWizard [28]. Peak picking was set to MS level 1 denoting MS^1^ scans, and the absolute peak intensity threshold was set to 0.001, suggesting the minimum intensity for a peak to be detected. Peak detection, retention time alignment, grouping, and gap filling were done using XCMS [29] and in-house scripts in R [30], with appropriate parameters. A two-group comparison between (1) QC and blanks and (2) QC and samples was made using the ‘diffreport’ function of XCMS. The QC vs. blanks list was used to identify *m/z* features that were non-significant (α = 0.05) between the two groups. These features correspond to background noise contributed by blanks and were eliminated from the QC vs. samples list of features. The QC vs. samples list was further cleaned by visually examining all extracted ion chromatograms (EICs) generated by diffreport and deleting *m/z* features that represented background noise. 

The filtered matrix was then subjected to run-order normalization by a QC based robust LOESS signal correction (QC-RLSC) [31], and run-order effects were evaluated before and after normalization. Relative standard deviation (RSD) [32] of all *m/z* features in the QC was determined and features in the normalized data matrix with RSD > 0.3 were eliminated. The resultant data matrix was used for downstream statistical analyses lipid ions identified by LipidSearch^™^ software for lipidomics.

#### 4.4.3. Lipid Annotation and Identification

Identification of lipids by LipidSearch™ was based on comparison of the ddMS^2^ data obtained from select samples with in silico calculations/database. Since peak detection and quantitation were computed by XCMS, LipidSearch results were exclusively used for identification. Default parameters were therefore used for LipidSearch quantitation. The quality of matches with the database is denoted by the alphabets ‘A’, ‘B’, ‘C’, and ‘D’. Only lipid species with grades A, B or C were used in this study. Grade A indicates lipids of which fatty acids and class are identified completely, B denotes lipids of which class and some fatty acids are identified, and C represents lipids of which class or fatty acids are identified. Potential fatty acid options and their tentative positions (e.g., *sn*-1, *sn*-2) are provided with respective scores (e.g., PE(16:0/18:1) + H for phosphatidylethanolamine with 16:0 and 18:1 fatty acids; Figure S1). While the exact position of fatty acids cannot be deduced via ddMS^2^ data, the m-score, a score calculated based on the number of matches with product ion peaks was used to denote lipid species with high reliability. Grade C species are presented as a sum of carbon atoms and their double bonds (e.g., PE(34:1) + H, thereby limiting interpretation of fatty acid information. 

To ensure correct identification of lipid species by the LipidSearch^TM^ software, one lipid species from each class was manually identified. Here, (i) the extracted ion chromatogram (EIC) of the parent ion was generated, and elemental composition was verified (± 10 ppm), (ii) principal product ions from analytical standards for the corresponding lipid species were acquired from the LipidMaps database (www.lipidmaps.org/resources/standards), and finally (iii) product ions from the raw file (ddMS^2^), analytical standard (LipidMaps), and LipidSearch^TM^ results were compared. An exemplar for PE(16:0/18:1) + H alone is demonstrated in Figure S1. Furthermore, Taguchi and Ishikawa [33] have reported 96–98% similarity between manual identification and LipidSearch^TM^ results in mouse brain and liver. The lipid classes used by LipidSearch are presented in Table S6. For this study, [M − H]^−^ and [M + HCOO]^−^ adducts, and [M + H]^+^ and [M + NH_4_]^+^ adducts were selected for annotation of negative and positive ionization modes, respectively. Finally, a retention time tolerance of 0.01 min, and mass error tolerance of ± 5 ppm was allowed. Results/identifications from individual MS^2^ files (LipidSearch results) were concatenated into a single table (one each for positive and negative ionization modes), and matched with the respective, normalized, peak intensity tables (XCMS results). 

#### 4.4.4. Chemometrics

Statistical analyses were performed using an online chemometric analysis suite, MetaboAnalyst ver 3.0 [34]. By default, MetaboAnalyst replaces all missing and zero values with half of the minimum positive value. A data scaling procedure, i.e., auto-scaling, where the data were normalized (mean-centered and divided by standard deviation of each variable) so that the features (peak intensities) are comparable was also carried out.

### 4.5. Human Milk and Infant Formula Composition

All analyses were conducted in duplicate.

#### 4.5.1. Fatty Acid Analyses

Fatty acids were extracted using hexane:propanol and the methyl esters formed and measured by GC-FID as per the method of Christie [35]. Briefly, to 2 mL of milk, 4 mL propanol, and 3 mL hexane was added and the sample was vigorously mixed. The hexane layer was removed and the milk sample re-extracted with a further 3 mL hexane and the hexane extract combined and evaporated to dryness. The dried extract was re-solubilized in 4 mL hexane and methylated with 0.1 mL of 0.5 M sodium methoxide in methanol. After 10 min the sample was neutralized with 5 μL glacial acetic acid, dried with CaCl_2_ for 60 min, and an aliquot placed in a 1.5 mL GC vial for GC-FID analysis.

GC analysis was performed using a Shimadzu GC-2010 plus (Shimadzu Corporation, Kyoto, Japan) equipped with a flame ionization detector (FID). Fatty acid methyl esters (FAMEs) were resolved (Restek RTX 2330 column, 105 m × 0.25 mm i.d, 0.20 μm film thickness; Restek Corporation, Bellefonte, PA, USA) with thermal gradient elution of the column oven at an initial temperature of 75 °C for 5 min, which was increased to 250 °C at a rate of 25 °C/min and held for 6 min. The carrier gas was hydrogen with a linear velocity of 50 cm/sec (3.05 mL/min). The injection volume was 1 μL, with a split ratio of 80:1. The injector temperature was 260 °C and the detector temperature was 300 °C. Fatty acids were reported as a percentage of the total after peak areas were corrected for detector response using theoretical FID response factors. The equations for generating the response and conversion factors to quantify individual fatty acids from the FAMEs were obtained from American Oil Chemists’ Society (AOCS Ce 1f-96, Ce 1h-05 and Ce 1i-07).

#### 4.5.2. PL Analysis of Milk Samples

Milk lipids were extracted using Folch method and analysis was as described by Reis, Roy [36]. Briefly, to 0.5 mL of milk sample 10 mL of chloroform:methanol (2:1, *v*/*v*) was added. The whole mixture was agitated for 20 min and then centrifuged. The liquid phase was recovered. Solvent partition was achieved by addition of 2 mL of 0.9% NaCl and gentle inversion 3 to 4 times, followed by centrifugation. The upper phase was removed. The lower phase was rinsed twice with methanol:water (1:1, *v*/*v*), without mixing the whole preparation. After centrifugation, the lower phase was removed and dried. The amount of PL in this extract were quantified using HPLC-ELSD (Shimadzu, Kyoto, Japan) equipped with two LC-10 ADvp pumps, a SCL-10 ADvp gradient system, a DGU-14 ADvp module degasser, and SIL-10ADvp autosampler. The analytical column was a YMC – pack PVA-SIL-NP column (250 × 4.6 mm, 5µm). The chromatographic separation was carried out using a linear binary gradient according to the following scheme: t0 = 0% B, t30 = 40% B, and isocratic conditions (40% B) for 1 min. Eluent A consisted of chloroform:isopropanol:triethylamine:acetic acid (75:25:0.08:1.0, *v*/*v*/*v*/*v*) and eluent B was methanol:water:triethylamine:acetic acid (95:5:0.08:1.0, *v*/*v*/*v*/*v*). The flow rate of the eluent was 1.0 mL/min. An ELSD-LT II Shimadzu model ELSD was used for detection; the pressure of nebulizer gas (nitrogen) was maintained at 350 kPa and the drift tube temperature was set at 50 °C. Identification of PLs and sphingomyelins was carried out by comparison with the retention time of pure standards. Calibration curves for each compound were calculated from area values obtained by injecting different volumes of chloroform solution containing phosphatidylethanolamine, phosphatidylinositol, phosphatidylcholine, phosphatidylserine, and sphingomyelin (SM). The PL standards were supplied by Sigma-Aldrich.

#### 4.5.3. TAG Analyses

Lipids were extracted according to published methods [37,38], and as described above (PL analysis of milk samples). The lipid extract was resolubilized in toluene and a 1 μL aliquot analyzed by GC-MS. The GC-MS (Shimadzu QP-2010, Shimadzu, Japan) was programmed to operate in full-scan mode scanning from 50–1000 *m/z* at 5 Hz (5000 Da/sec). The column (Restek RX 65TG, 30 m × 0.25 mm i.d, 0.10 µm film thickness,) was held at 70 °C for 2.5 min, then heated at 5 °C/min to 370 °C and held for 20 min. Helium was used as carrier gas at a flow rate of 1.75 mL/min (48.5 cm/s linear velocity), and a split ratio of 15:1. The injector temperature was 350 °C. Putative species for TAGs were determined as described by Teng, Reis [39].

### 4.6. Ethics Statement

Collection of human milk was performed with approved consent by participants, Approval no. XHEC-C-2012-024, Xinhua Hospital Ethics Committee Affiliated to Shanghai Jiào tong University School of Medicine. The animal study was conducted under the oversight of the AgResearch Grasslands Animal Ethics Committee (approval number AEC13099; Palmerston North, New Zealand) in accordance with the New Zealand Animal Welfare Act 1999.

## 5. Conclusions

Our data show that feeding human milk or infant formula to weanling rats for a period of 28 days can lead to differences in total brain SM concentrations. These results are not explained by simple differences in FA concentrations of the human milk or infant formula fed to the rats, indicating the presence complex mechanisms governing the distribution of lipids in the brain. In future studies, a more detailed examination of the FA spatial distribution may shed more light on the functional differences of human milk and infant formula on brain structure and function.

## Figures and Tables

**Table 1:** Fatty acid profiles of human milk and infant formula samples

Fatty Acid (% of Total FA)	Human Milk	Infant Formula
C4:0		0.38
C6:0		0.34
C8:0		1.26
C10:0	1.13	1.21
C12:0	4.26	7.25
C14:0	4.02	4.10
C14:1		0.12
C15:0	0.08	0.18
C16:0	22.40	23.78
C16:1	1.87	0.27
C17:0	0.18	0.14
C18:0	5.96	4.68
C18:1 n9	30.41	34.03
C18:1 n11	1.75	0.74
C18:2 n6	23.44	17.52
C18:3 n6		0.17
C18:3 n3	1.53	1.80
C20:0	0.19	0.29
C20:1	0.44	0.22
C20:2	0.43	
C20:3 n3	0.17	0.24
C20:4 n6	0.53	0.48
C20:5 n3		0.06
C22:5	0.16	
C22:6 n3	0.46	0.28
C24:0		0.18
C24:1	0.05	

Fatty acids are expressed as a percentage of total fatty acids.

**Table 2:** Triacylglycerol (TAG) profile of human milk and infant formula samples

Total Carbon Chain Length (% Total TAG)	Human Milk	Infant Formula	Putative Species
C30	0.9	0.82	C14:0C12:0C4:0/C12:0C14:0C4:0/ C8:0C12:0C10:0/ C10:0C16:0C4:0/ C8:0C18:0C4:0/ C10:0C18:1C4:0/ C10:0C18:2C6:0
C32	0.56	2.21	C16:0C10:0C6:0/C10:0C16:0C6:0/ C14:0C14:0C4:0/ C16:0C12:0C4:0/C12:0C16:0C4:0/ C10:0C18:0C4:0
C34	0.26	8.23	C16:1C14:0C4:0/ C18:1C10:0C6:0/ C16:0C14:1C4:0/ C12:0C12:0C10:0/C12:0C10:0C12:0/ C10:0C18:0C6:0/ C14:0C8:0C12:0/C8:0C14:0C12:0/ C18:0C12:0C4:0/C12:0C18:0C4:0/ C16:0C14:0C4:0
C36	1.06	12.3	C18:2C14:0C4:0/ C8:0C18:1C10:0/ C18:1C14:0C4:0/C14:0C18:1C4:0/ C16:0C14:0C6:0/ C16:0C16:0C4:0
C38	1.78	15.13	C18:1C16:1C4:0/ C18:2C16:0C4:0/ C18:1C10:0C10:0/C10:0C18:1C10:0/ C18:1C16:0C4:0/C16:0C18:1C4:0/ C12:0C16:0C10:0/ C16:0C16:0C6:0/ C16:0C18:0C4:0
C40	3.11	11.88	C18:2C18:1C4:0/ C18:1C18:1C4:0/ C18:1C12:0C10:0/ C18:1C16:0C6:0/C16:0C18:1C6:0/ C18:0C18:1C4:0/ C16:0C14:0C10:0/ C16:0C18:0C6:0/C18:0C16:0C6:0
C42	5.48	8.79	C18:1C18:1C6:0/ C14:1C14:0C14:0/C14:0C14:1C14:0/ C18:1C10:0C14:0/C10:0C18:1C14:0/ C18:1C16:0C8:0/ C18:0C18:1C6:0/ C16:0C12:0C14:0/ C16:0C10:0C16:0/C16:0C16:0C10:0
C44	9.01	5.74	C10:0C16:0C18:3/ C18:1C8:0C18:1/C18:1C18:1C8:0/ C16:0C10:0C18:2/ C16:0C10:0C18:1/ C14:0C14:0C16:0/C14:0C16:0C14:0/ C18:0C10:0C16:0
C46	13.15	4.49	C16:0C12:0C18:3/ C14:1C14:0C18:1/C14:0C14:1C18:1/ C14:0C14:0C18:2/C14:0C18:2C14:0/ C16:0C12:0C18:2/ C16:0C12:0C18:1/ C14:0C16:1C16:0/ C18:0C10:0C18:1
C48	12.45	3.08	C16:0C14:0C18:3/ C16:0C14:0C18:2/ C16:0C14:1C18:1/ C16:0C14:0C18:1/ C18:0C14:0C16:0
C50	19.5	7.3	C18:1C14:0C18:2/ C16:0C16:1C18:2/ C18:1C14:0C18:1/C14:0C18:1C18:1/ C16:0C16:1C18:1/ C16:0C16:0C18:1/C16:0C18:1C16:0/ C16:0C18:0C16:0/C16:0C16:0C18:0/
C52	30.98	16.82	C16:0C18:2C18:2/C18:2C16:0C18:2/ C18:1C16:0C18:2/ C16:0C18:1C18:1/C18:1C16:0C18:1/ C18:0C16:0C18:1/ C18:0C16:0C18:0/C18:0C18:0C16:0
C54	1.76	3.21	C18:1C18:1C18:2/C18:1C18:2C18:1/ C18:1C18:1C18:1/ C18:0C18:2C18:1/ C18:1C18:0C18:1/C18:0C18:1C18:1/ C18:0C18:1C18:0/C18:0C18:0C18:1/ C18:0C18:0C18:0

TAGs are expressed as a percentage of total fatty acids.

**Table 3:** Distribution and absolute concentrations (mg/100 mL ± SE) of different phospholipid (PL) classes in human milk and infant formula samples

Phospholipid (mg/100 mL)	Human Milk	Infant Formula
PI	1.13 ± 0.16	3.83 ± 0.56
PE	2.62 ± 0.26	5.10 ± 1.17
PS	1.46 ± 0.18	3.66 ± 0.22
PC	3.27 ± 0.53	8.40 ± 0.64
SM	3.39 ± 0.19	7.08 ± 1.30
Total PL	11.87 ± 1.32	28.11 ± 2.14

PI—Phosphatidylinositol; PE—Phosphatidylethanolamine; PS—Phosphatidylserine; PC—Phosphatidylcholine; SM—Sphingomyelin.

## Supplementary Materials

The following are available online at https://www.mdpi.com/2218-1989/9/11/253/s1, Table S1. Tentative product/fragment ions of PE(16:0/18:1) + H and their corresponding observed *m/z* values generated by LipidSearch^TM^; Table S2. Putative combinations of fatty acids and adducts corresponding to PE(34:1) and their m-scores generated by LipidSearch^TM^; Table S3. List of 163 lipid species identified in the current study by LipidSearch^TM^ software (based on ddMS^2^) and matched with the final data matrix (XCMS) in positive and negative ionization modes, with average normalized peak intensities ± SE in brain samples from rats fed human milk or infant formula (*n* = 12); Table S4. Lipid species that were significantly different in the brain of rats fed human milk or infant formula (*n* = 12) based on percentage contribution to respective class; Table S5. Nutritional composition analysis of human milk and infant formula samples; Table S6. List of lipids identified by LipidSearch^™^ software; Figure S1. Identification of PE(16:0/18:1) + H where (A) shows an EIC corresponding to the parent ion *m/z* 718.5392, (B) shows elemental composition of *m/z* 718.5392 corresponding to the molecular formula of PE(16:0/18:1) + H, (C) shows the ddMS2 spectrum for *m/z* 718.54 @ CE of 30 eV, (D) shows principal product ions of an analytical standard of PE(16:0/18:1) + H from the LipidMaps database, and (E) shows the theoretical mass spectrum of MS2 product ions of *m/z* 718.5392.
