# Supplementary material for: Lipidomics of Brain Tissues in Rats Fed Human Milk from Chinese Mothers or Commercial Infant Formula
Source: Metabolites. 2019 Oct 28;9(11):253. doi: 10.3390/metabo9110253 (PMC6918239; doi:10.3390/metabo9110253)
Supplement: Supplementary file 1 [file metabolites-09-00253-s001.pdf]

*Article*

# Lipidomics of Brain Tissues in Rats Fed Human Milk from Chinese Mothers or Commercial Infant Formula

**Miya Su [1,†], Arvind K. Subbaraj [2,†,*], Karl Fraser [2,3,4], Xiaoyan Qi [1], Hongxin Jia [1], Wenliang Chen [1], Mariza Gomes Reis [2], Mike Agnew [2], Li Day [2], Nicole C. Roy [2,3,4] and Wayne Young [2,3,4]**

[1] State Key Laboratory of Dairy Biotechnology, Dairy Research Institute, Bright Dairy and Food Co. Ltd., Shanghai 200436, China; shumiya@brightdairy.com (M.S.); qixiaoyan@brightdairy.com (X.Q.); jiahongxin@brightdairy.com (H.J.); chenwenliang@brightdairy.com (W.C.)

[2] AgResearch Ltd., Grasslands Research Centre, Palmerston North 4442, New Zealand; Arvind.Subbaraj@agresearch.co.nz (A.K.S.); Karl.Fraser@agresearch.co.nz (K.F.); Mariza.GomesReis@agresearch.co.nz (M.G.R.); Michael.Agnew@agresearch.co.nz (M.A.); Li.Day@agresearch.co.nz (L.D.); roynnz33@gmail.com (N.C.R.); Wayne.Young@agresearch.co.nz (W.Y.)

[3] Riddet Institute, Massey University, Palmerston North 4474, New Zealand

[4] High-Value Nutrition National Science Challenge, Auckland 1023, New Zealand

† These authors contributed equally to this work

* Correspondence: Arvind.Subbaraj@agresearch.co.nz; Tel.: +64-6-3518060

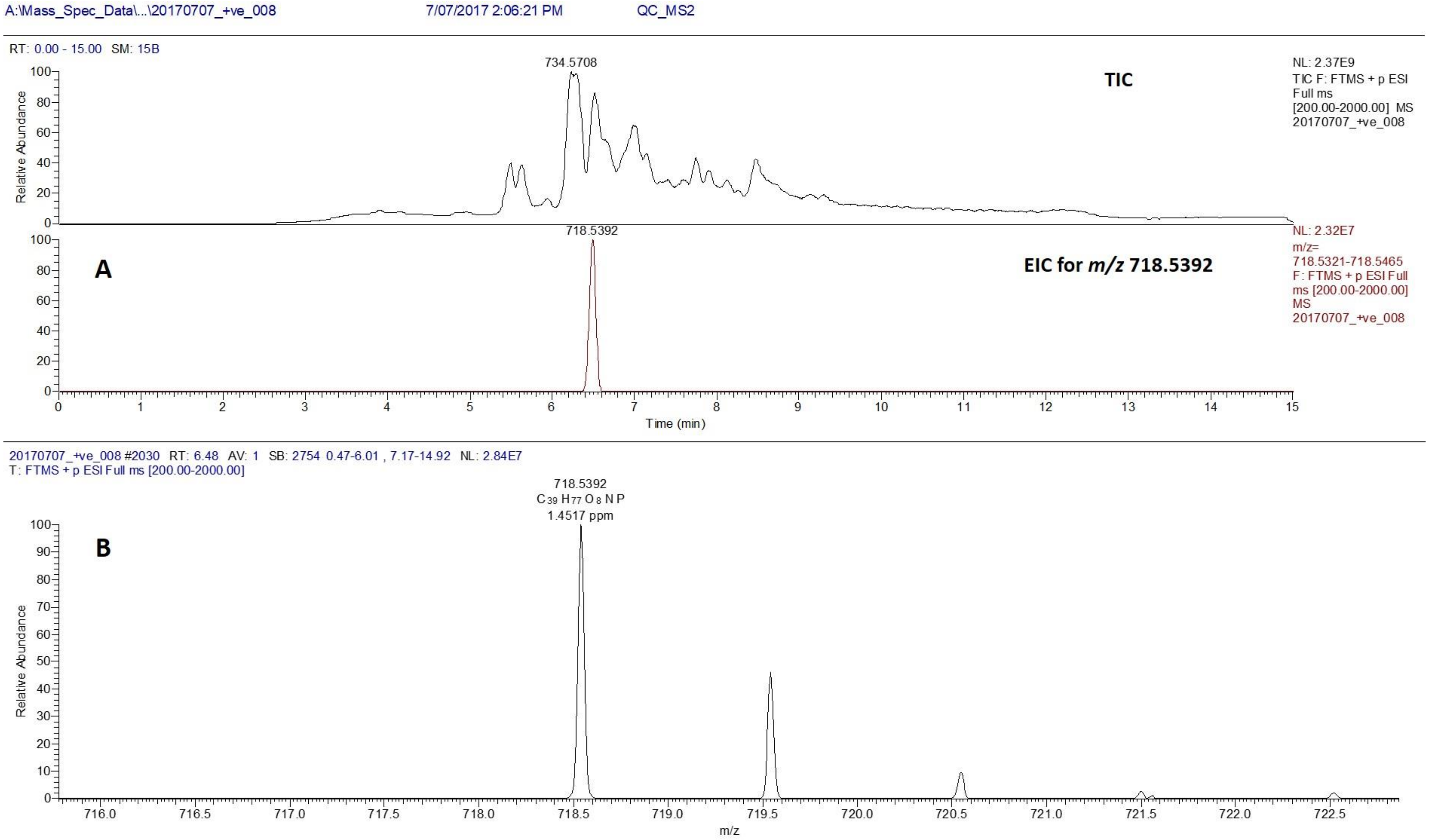
A:\Mass_Spec_Data\...\20170707_+ve_008
7/07/2017 2:06:21 PM
QC_MS2
RT: 0.00 - 15.00 SM: 15B
734.5708
TIC
NL: 2.37E9
TIC F: FTMS + p ESI Full ms [200.00-2000.00] MS 20170707_+ve_008
718.5392
A
EIC for m/z 718.5392
NL: 2.32E7
m/z= 718.5321-718.5465 F: FTMS + p ESI Full ms [200.00-2000.00] MS 20170707_+ve_008
Relative Abundance
Time (min)
20170707_+ve_008 #2030 RT: 6.48 AV: 1 SB: 2754 0.47-6.01 , 7.17-14.92 NL: 2.84E7
T: FTMS + p ESI Full ms [200.00-2000.00]
718.5392
C39 H77 O8 N P
1.4517 ppm
B
m/z

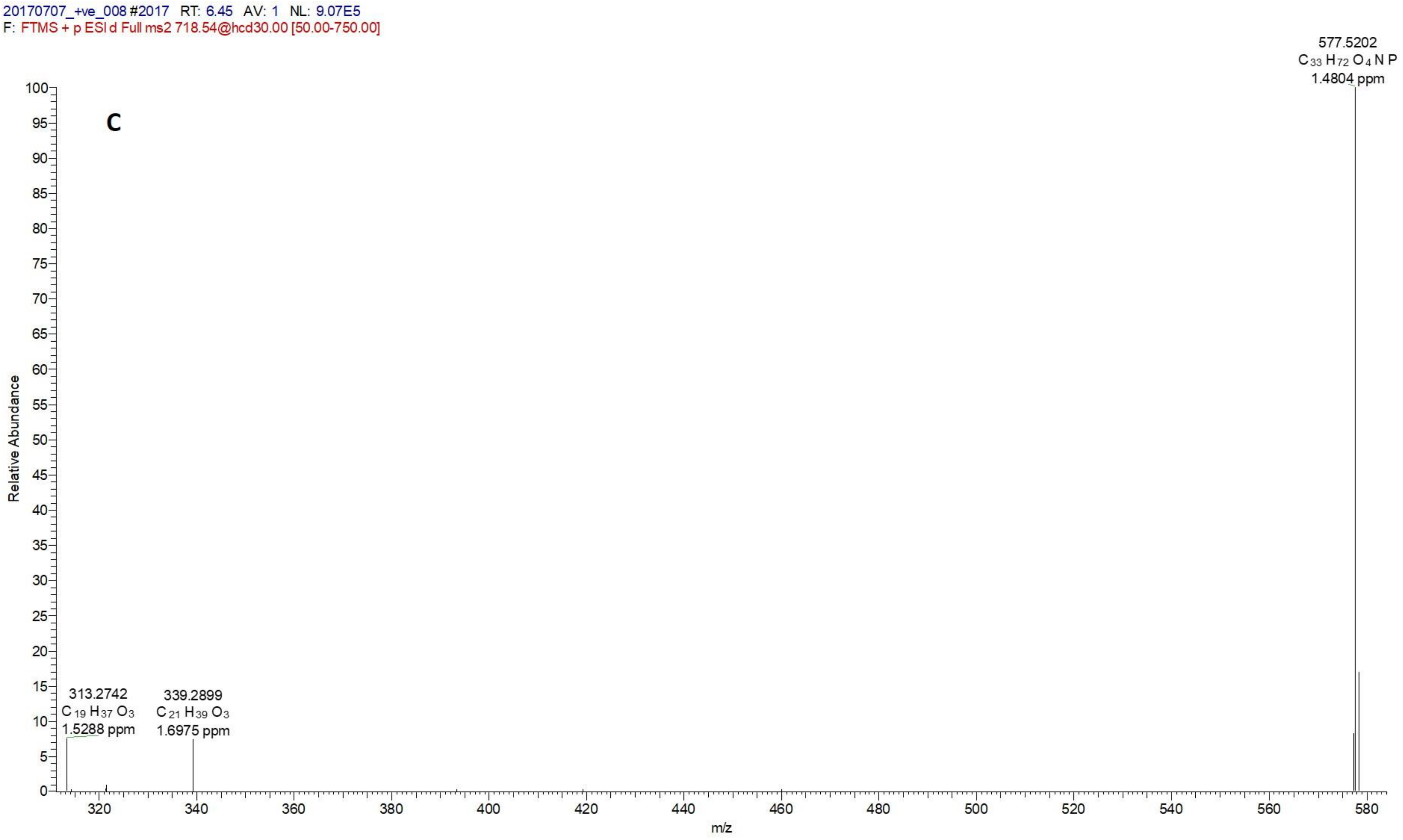
20170707_+ve_008 #2017 RT: 6.45 AV: 1 NL: 9.07E5
F: FTMS + p ESI d Full ms2 718.54@hcd30.00 [50.00-750.00]
C
577.5202
C 33 H 72 O 4 N P
1.4804 ppm
313.2742
C 19 H 37 O 3
1.5288 ppm
339.2899
C 21 H 39 O 3
1.6975 ppm
Relative Abundance
m/z

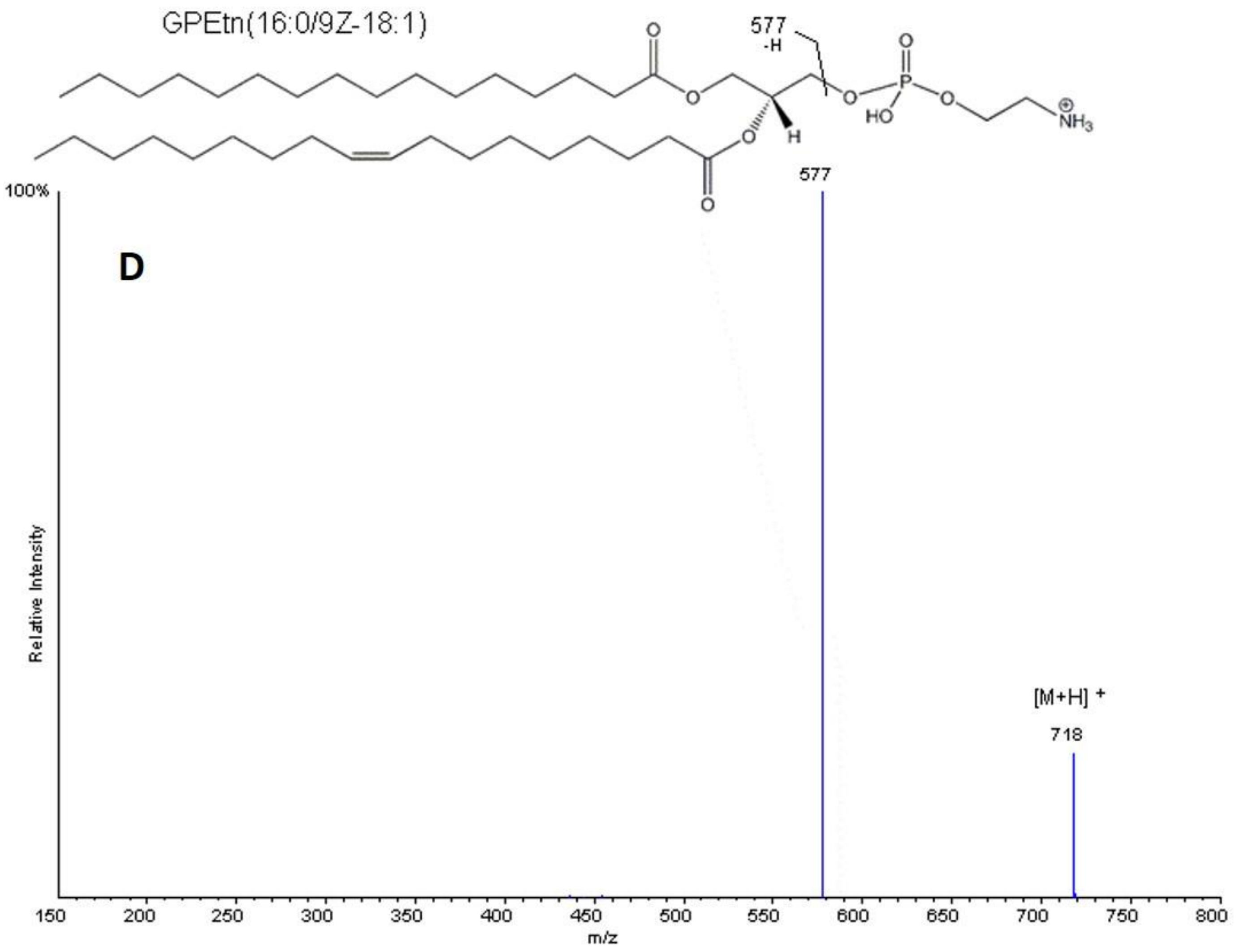
GPEtn(16:0/9Z-18:1)
577
-H
HO
NH3
H
577
100%
D
Relative Intensity
[M+H] +
718
150
200
250
300
350
400
450
500
550
600
650
700
750
800
m/z

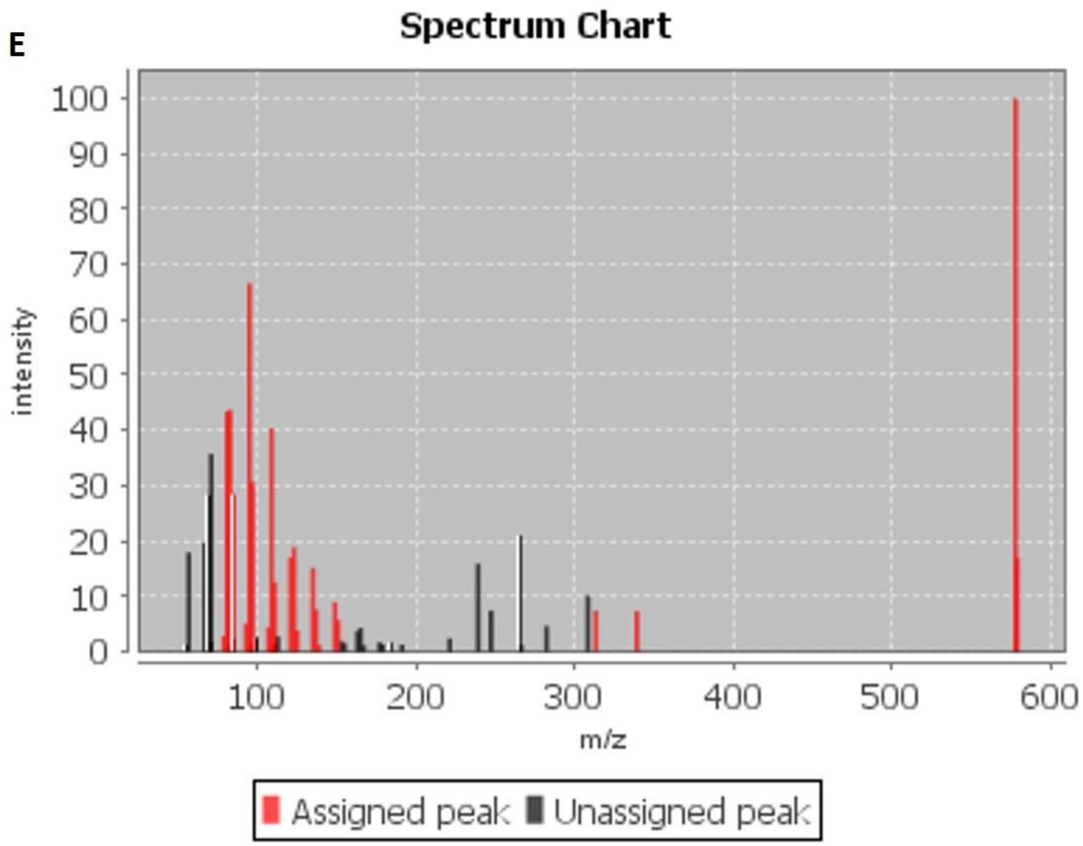


**Figure S1.** Identification of PE(16:0/18:1)+H where **(A)** shows an EIC corresponding to the parent ion *m/z* 718.5392, **(B)** shows elemental composition of *m/z* 718.5392 corresponding to the molecular formula of PE(16:0/18:1)+H, **(C)** shows the ddMS2 spectrum for *m/z* 718.54 @ CE of 30 eV, **(D)** shows principal product ions of an analytical standard of PE(16:0/18:1)+H from the LipidMaps database, and **(E)** shows the theoretical mass spectrum of MS2 product ions of *m/z* 718.5392.

**Table S1.** Tentative product/fragment ions of PE(16:0/18:1)+H and their corresponding observed *m/z* values generated by LipidSearch™.

| **Observed *m/z*** | **Tentative product/fragment ions** |
|---|---|
| 79.0548 | $C_6H_7$ |
| 81.0705 | $C_6H_9$ |
| 83.0862 | $C_6H_{11}$ |
| 85.1018 | $C_6H_{13}$ |
| 93.0704 | $C_7H_9$ |
| 95.0861 | $C_7H_{11}$ |
| 97.1017 | $C_7H_{13}$ |
| 107.086 | $C_8H_{11}$ |
| 109.1016 | $C_8H_{13}$ |
| 111.1172 | $C_8H_{15}$ |
| 121.1015 | $C_9H_{13}$ |
| 123.1171 | $C_9H_{15}$ |
| 125.1328 | $C_9H_{17}$ |
| 135.117 | $C_{10}H_{15}$ |
| 137.1325 | $C_{10}H_{17}$ |
| 139.1483 | $C_{10}H_{19}$ |
| 149.1327 | $C_{11}H_{17}$ |
| 151.1484 | $C_{11}H_{19}$ |
| 313.2741 | (16:0)-OH |
| 339.2897 | (18:1)-OH |
| 577.5199 | NL[PE] |

NL[PE] refers to the neutral loss of *m/z* 141 diagnostic of PE

**Table S2.** Putative combinations of fatty acids and adducts corresponding to PE(34:1) and their m-scores generated by LipidSearch™.

| Lipid ion | m-score* |
|---|---|
| PE(16:0/18:1)+H | 29.8 |
| PE(18:1/16:0)+H | 29.8 |
| PE(10:0/24:1)+H | 15.1 |
| PE(12:0/22:1)+H | 15.1 |
| PE(14:0/20:1)+H | 15.1 |
| PE(15:1/19:0)+H | 15.1 |
| PE(16:1/18:0)+H | 15.1 |
| PE(17:1/17:0)+H | 15.1 |
| PE(18:0/16:1)+H | 15.1 |
| PE(19:1/15:0)+H | 15.1 |
| PE(20:0/14:1)+H | 15.1 |
| PE(20:1/14:0)+H | 15.1 |
| PE(22:1/12:0)+H | 15.1 |
| PE(24:1/10:0)+H | 15.1 |
| PE(26:1/8:0)+H | 15.1 |
| PE(16:0e/19:4)+Li | 15.1 |
| PE(16:0p/19:3)+Li | 15.1 |
| PE(16:1e/19:3)+Li | 15.1 |
| PE(16:1p/19:2)+Li | 15.1 |
| PE(18:0p/17:3)+Li | 15.1 |

*The score calculated based on the number of matches with product ion peaks in the spectrum.

**Table S3.** List of 163 lipid species identified in the current study by LipidSearch software (based on ddMS[2]) and matched with the final data matrix (XCMS) in positive and negative ionisation modes, with average normalised peak intensities ± SE in brain samples from rats fed human milk or infant formula (*n*=12).

| Lipid Ion | *m/z* | RT (secs) | Ion Formula | Class | Fatty Acid | Average peak intensity ± SE | |
|---|---|---|---|---|---|---|---|
| | | | | | | Human milk | Infant formula |
| **Positive ionisation mode** | | | | | | | |
| **Ceramides** | | | | | | | |
| Cer(d18:1/16:0)+H | 538.5194 | 381.28 | $C_{34}H_{68}O_3N$ | Cer | (d18:1/16:0) | 23390237 ± 849340 | 22968114 ± 849277 |
| Cer(d18:2/18:0)+H | 564.535 | 386.52 | $C_{36}H_{70}O_3N$ | Cer | (d18:2/18:0) | 70383939 ± 3456659 | 71824796 ± 3092380 |
| Cer(d18:1/18:0)+H | 566.5507 | 427.14 | $C_{36}H_{72}O_3N$ | Cer | (d18:1/18:0) | 471871680 ± 27081731 | 468352547 ± 18724515 |
| Cer(d20:1/18:0)+H | 594.582 | 472 | $C_{38}H_{76}O_3N$ | Cer | (d20:1/18:0) | 46137740 ± 1586564 | 41710459 ± 2727779 |
| Cer(d18:1/22:0)+H | 622.6133 | 515.04 | $C_{40}H_{80}O_3N$ | Cer | (d18:1/22:0) | 7542913 ± 384422 | 6753847 ± 453905 |
| Cer(d18:1/24:1)+H | 648.6289 | 512.34 | $C_{42}H_{82}O_3N$ | Cer | (d18:1/24:1) | 47623616 ± 2526683 | 43930662 ± 2659910 |
| Cer(d18:1/24:0)+H | 650.6446 | 555.02 | $C_{42}H_{84}O_3N$ | Cer | (d18:1/24:0) | 7193849 ± 424658 | 6287841 ± 405475 |
| CerG1(d18:1/18:0)+H | 728.6035 | 386.54 | $C_{42}H_{82}O_8N$ | CerG1 | (d18:1/18:0) | 47372243 ± 1804599 | 42712717 ± 2622198 |
| CerG1(d18:1/18:0+O)+H | 744.5984 | 375.48 | $C_{42}H_{82}O_9N$ | CerG1 | (d18:1/18:0+O) | 21766753 ± 1159251 | 17850453 ± 1365347 |
| CerG1(d18:1/20:0+O)+H | 772.6297 | 417.6 | $C_{44}H_{86}O_9N$ | CerG1 | (d18:1/20:0+O) | 21766753 ± 1159251 | 17850453 ± 1365347 |

| | | | | | | | |
|---|---|---|---|---|---|---|---|
| CerG1(d38:1)+H | 756.6348 | 431 | $C_{44}H_{86}O_8N$ | CerG1 | (d38:1) | 23059911 ± 1296903 | 19095985 ± 1694169 |
| CerG1(d18:1/22:1)+H | 782.6504 | 464.2 | $C_{46}H_{88}O_8N$ | CerG1 | (d18:1/22:1) | 14325563 ± 647307 | 11579016 ± 920522 |
| CerG1(d18:1/22:0)+H | 784.6661 | 472.5 | $C_{46}H_{90}O_8N$ | CerG1 | (d18:1/22:0) | 32200872 ± 1694967 | 26462477 ± 2670847 |
| CerG1(d40:0+O)+H | 802.6767 | 480.66 | $C_{46}H_{92}O_9N$ | CerG1 | (d40:0+O) | 22680879 ± 1843054 | 17708949 ± 2024657 |
| CerG1(d18:1/24:2)+H | 808.6661 | 462.38 | $C_{48}H_{90}O_8N$ | CerG1 | (d18:1/24:2) | 12171501 ± 575191 | 9687452 ± 811384 |
| CerG1(d18:2/24:1)+H | 808.6661 | 433.88 | $C_{48}H_{90}O_8N$ | CerG1 | (d18:2/24:1) | 5069266 ± 242880 | 4440493 ± 374297 |
| CerG1(d18:1/24:1)+H | 810.6817 | 472.22 | $C_{48}H_{92}O_8N$ | CerG1 | (d18:1/24:1) | 166696157 ± 8407145 | 135403339 ± 13946084 |
| CerG1(d42:1)+H | 812.6974 | 516.16 | $C_{48}H_{94}O_8N$ | CerG1 | (d42:1) | 121747728 ± 7099915 | 104824272 ± 9976147 |
| CerG1(d18:0/24:1)+H | 812.6974 | 485.84 | $C_{48}H_{94}O_8N$ | CerG1 | (d18:0/24:1) | 7223754 ± 374450 | 5339250 ± 655133 |
| CerG1(d18:0/24:0)+H | 814.713 | 530.82 | $C_{48}H_{96}O_8N$ | CerG1 | (d18:0/24:0) | 11089637 ± 495319 | 8292721 ± 894933 |
| CerG1(d42:0+O)+H | 830.708 | 521.86 | $C_{48}H_{96}O_9N$ | CerG1 | (d42:0+O) | 56064641 ± 2745368 | 41254337 ± 3995143 |
| CerG1(d18:1/26:1)+H | 838.713 | 512.7 | $C_{50}H_{96}O_8N$ | CerG1 | (d18:1/26:1) | 10855874 ± 673048 | 9145632 ± 793104 |
| CerG1(d44:1)+H | 840.7287 | 554.86 | $C_{50}H_{98}O_8N$ | CerG1 | (d44:1) | 7331391 ± 511900 | 6021735 ± 514105 |
| **Diacylglycerol** | | | | | | | |
| DG(18:0/16:0)+$NH_4$ | 614.5718 | 502.76 | $C_{37}H_{76}O_5$ N | DG | (18:0/16:0) | 14683932 ± 442610 | 13057723 ± 589684 |
| DG(18:0/18:1)+$NH_4$ | 640.5875 | 506.04 | $C_{39}H_{78}O_5N$ | DG | (18:0/18:1) | 10987111 ± 849163 | 9072100 ± 1031855 |

| | | | | | | | |
|---|---|---|---|---|---|---|---|
| DG(18:0/18:0)+$NH_4$ | 642.6031 | 541.98 | $C_{39}H_{80}O_5N$ | DG | (18:0/18:0) | 38458623 ± 1660750 | 35287483 ± 1280747 |
| DG(18:0/20:4)+$NH_4$ | 662.5718 | 461.2 | $C_{41}H_{76}O_5N$ | DG | (18:0/20:4) | 27261396 ± 2594638 | 24747622 ± 2644507 |
| **Phosphatidylethanolamine** | | | | | | | |
| PE(16:0p/16:0)+H | 676.5276 | 407.82 | $C_{37}H_{75}O_7NP$ | PE | (16:0p/16:0) | 5297000 ± 223815 | 4618677 ± 232877 |
| PE(16:0p/18:1)+H | 702.5432 | 412.7 | $C_{39}H_{77}O_7NP$ | PE | (16:0p/18:1) | 229567636 ± 9557431 | 198310814 ± 13014100 |
| PE(18:0p/16:0)+H | 704.5589 | 451.78 | $C_{39}H_{79}O_7NP$ | PE | (18:0p/16:0) | 13159539 ± 550477 | 11404356 ± 658703 |
| PE(16:0e/18:1)+H | 704.5589 | 417.32 | $C_{39}H_{79}O_7NP$ | PE | (16:0e/18:1) | 31287163 ± 900958 | 26701141 ± 1669861 |
| PE(16:0/18:1)+H | 718.5381 | 387.28 | $C_{39}H_{77}O_8NP$ | PE | (16:0/18:1) | 92758254 ± 2504884 | 87335358 ± 3773007 |
| PE(18:0/16:0)+H | 720.5538 | 426.64 | $C_{39}H_{79}O_8NP$ | PE | (18:0/16:0) | 16058191 ± 694886 | 14978929 ± 715916 |
| PE(16:0p/20:4)+H | 724.5276 | 367.12 | $C_{41}H_{75}O_7NP$ | PE | (16:0p/20:4) | 103670452 ± 5357121 | 100137061 ± 4827411 |
| PE(18:1p/18:1)+H | 728.5589 | 414.84 | $C_{41}H_{79}O_7NP$ | PE | (18:1p/18:1) | 303861858 ± 11694492 | 261609275 ± 19165290 |
| PE(18:0p/18:1)+H | 730.5745 | 452.26 | $C_{41}H_{81}O_7NP$ | PE | (18:0p/18:1) | 197605140 ± 10075566 | 162970118 ± 12751155 |
| PE(18:0e/18:1)+H | 732.5902 | 462.42 | $C_{41}H_{83}O_7NP$ | PE | (18:0e/18:1) | 25168614 ± 1166438 | 19952740 ± 1647797 |
| PE(16:0/20:4)+H | 740.5225 | 345.86 | $C_{41}H_{75}O_8NP$ | PE | (16:0/20:4) | 44849131 ± 2441902 | 45055266 ± 2221239 |
| PE(18:0/18:1)+H | 746.5694 | 431.88 | $C_{41}H_{81}O_8NP$ | PE | (18:0/18:1) | 129706948 ± 4988478 | 111512912 ± 6619286 |
| PE(16:0p/22:6)+H | 748.5276 | 353.7 | $C_{43}H_{75}O_7NP$ | PE | (16:0p/22:6) | 164904737 ± 9020185 | 154992902 ± 7949434 |

| | | | | | | | |
|---|---|---|---|---|---|---|---|
| PE(18:1p/20:1)+H | 756.5902 | 452.3 | $C_{43}H_{83}O_7NP$ | PE | (18:1p/20:1) | 49049516 ± 3583071 | 36536204 ± 3374387 |
| PE(18:0p/20:1)+H | 758.6058 | 491.34 | $C_{43}H_{85}O_7NP$ | PE | (18:0p/20:1) | 33159888 ± 1940875 | 26517580 ± 2609547 |
| PE(16:0/22:6)+H | 764.5225 | 335.18 | $C_{43}H_{75}O_8NP$ | PE | (16:0/22:6) | 240338921 ± 11822540 | 236126020 ± 12061883 |
| PE(18:1/20:4)+H | 766.5381 | 350.64 | $C_{43}H_{77}O_8NP$ | PE | (18:1/20:4) | 50382385 ± 2540264 | 47897491 ± 2319392 |
| PE(18:0/20:3)+H | 770.5694 | 408.54 | $C_{43}H_{81}O_8NP$ | PE | (18:0/20:3) | 8264646 ± 392065 | 7540929 ± 447905 |
| PE(18:1p/22:6)+H | 774.5432 | 356.76 | $C_{45}H_{77}O_7NP$ | PE | (18:1p/22:6) | 60366085 ± 3482997 | 55840385 ± 2930860 |
| PE(18:0/20:1)+H | 774.6007 | 470.12 | $C_{43}H_{85}O_8NP$ | PE | (18:0/20:1) | 12210565 ± 694558 | 9356541 ± 923806 |
| PE(18:0p/22:6)+H | 776.5589 | 399.84 | $C_{45}H_{79}O_7NP$ | PE | (18:0p/22:6) | 471166520 ± 16679831 | 449560071 ± 21016084 |
| PE(18:1/22:6)+H | 790.5381 | 337.32 | $C_{45}H_{77}O_8NP$ | PE | (18:1/22:6) | 57122632 ± 3073425 | 55167080 ± 2665150 |
| PE(18:0/22:6)+H | 792.5538 | 380.92 | $C_{45}H_{79}O_8NP$ | PE | (18:0/22:6) | 740366682 ± 20173629 | 710290814 ± 27968280 |
| PE(18:0/22:5)+H | 794.5694 | 404 | $C_{45}H_{81}O_8NP$ | PE | (18:0/22:5) | 27531820 ± 1259669 | 23731642 ± 1059150 |
| PE(18:0/22:4)+H | 796.5851 | 421.82 | $C_{45}H_{83}O_8NP$ | PE | (18:0/22:4) | 105666250 ± 3876693 | 98916103 ± 4576959 |
| **Sphingomyelins** | | | | | | | |
| SM(d34:1)+H | 703.5749 | 325.24 | $C_{39}H_{80}O_6N_2P$ | SM | (d34:1) | 50764155 ± 2154145 | 49620160 ± 3589264 |
| SM(d36:2)+H | 729.5905 | 327.32 | $C_{41}H_{82}O_6N_2P$ | SM | (d36:2) | 53108203 ± 1908169 | 49520389 ± 2910627 |
| SM(d36:1)+H | 731.6062 | 370.2 | $C_{41}H_{84}O_6N_2P$ | SM | (d36:1) | 595682085 ± 14313611 | 568798967 ± 25111132 |

| | | | | | | | |
|---|---|---|---|---|---|---|---|
| SM(d36:0)+H | 733.6218 | 386.58 | $C_{41}H_{86}O_6N_2P$ | SM | (d36:0) | 18506329 ± 702414 | 16668164 ± 830403 |
| SM(d38:1)+H | 759.6375 | 413.62 | $C_{43}H_{88}O_6N_2P$ | SM | (d38:1) | 95973869 ± 3355740 | 87308842 ± 5576838 |
| SM(d40:2)+H | 785.6531 | 416.98 | $C_{45}H_{90}O_6N_2P$ | SM | (d40:2) | 6802675 ± 417663 | 5215207 ± 495856 |
| SM(d40:1)+H | 787.6688 | 461 | $C_{45}H_{92}O_6N_2P$ | SM | (d40:1) | 17145771 ± 1023207 | 14014343 ± 1328073 |
| SM(d42:2)+H | 813.6844 | 455.84 | $C_{47}H_{94}O_6N_2P$ | SM | (d42:2) | 54735931 ± 3364245 | 44368485 ± 4268536 |
| SM(d42:1)+H | 815.7001 | 503.46 | $C_{47}H_{96}O_6N_2P$ | SM | (d42:1) | 17883630 ± 971659 | 15212062 ± 1275012 |
| **Phosphatidylcholine** | | | | | | | |
| PC(30:0)+H | 706.5381 | 328.84 | $C_{38}H_{77}O_8NP$ | PC | (30:0) | 268743924 ± 8859790 | 256007242 ± 13918027 |
| PC(32:1e)+H | 718.5745 | 395.32 | $C_{40}H_{81}O_7NP$ | PC | (32:1e) | 26293990 ± 1075666 | 25697721 ± 1125782 |
| PC(31:0)+H | 720.5538 | 348.6 | $C_{39}H_{79}O_8NP$ | PC | (31:0) | 18534715 ± 1118875 | 18851214 ± 1063688 |
| PC(32:0e)+H | 720.5902 | 397.54 | $C_{40}H_{83}O_7NP$ | PC | (32:0e) | 38882741 ± 1825303 | 35659901 ± 1668226 |
| PC(32:1)+H | 732.5538 | 331.72 | $C_{40}H_{79}O_8NP$ | PC | (32:1) | 251701301 ± 20931185 | 212956463 ± 11679169 |
| PC(32:0)+H | 734.5694 | 371.66 | $C_{40}H_{81}O_8NP$ | PC | (32:0) | 3244692833 ± 94953762 | 3187938505 ± 100957320 |
| PC(33:1)+H | 746.5694 | 354.24 | $C_{41}H_{81}O_8NP$ | PC | (33:1) | 16886949 ± 817511 | 16347720 ± 877657 |
| PC(34:1e)+H | 746.6058 | 402.26 | $C_{42}H_{85}O_7NP$ | PC | (34:1e) | 240570825 ± 9683638 | 203695792 ± 14676231 |
| PC(33:0)+H | 748.5851 | 393.22 | $C_{41}H_{83}O_8NP$ | PC | (33:0) | 26432770 ± 810261 | 26836742 ± 1395662 |

| | | | | | | | |
|---|---|---|---|---|---|---|---|
| PC(34:0e)+H | 748.6215 | 443.12 | $C_{42}H_{87}O_7NP$ | PC | (34:0e) | 10409157 ± 443582 | 9174314 ± 546908 |
| PC(34:1)+H | 760.5851 | 375.36 | $C_{42}H_{83}O_8NP$ | PC | (34:1) | 4739634496 ± 141951176 | 4599586978 ± 148321696 |
| PC(34:0)+H | 762.6007 | 415.52 | $C_{42}H_{85}O_8NP$ | PC | (34:0) | 820034285 ± 30492265 | 760280516 ± 36554903 |
| PC(34:2)+H | 758.5694 | 342 | $C_{42}H_{81}O_8NP$ | PC | (34:2) | 126887348 ± 8463306 | 142243771 ± 9037323 |
| PC(36:2p)+H | 770.6058 | 397.12 | $C_{44}H_{85}O_7NP$ | PC | (36:2p) | 19167159 ± 856519 | 16758444 ± 1223754 |
| PC(36:2e)+H | 772.6215 | 406.2 | $C_{44}H_{87}O_7NP$ | PC | (36:2e) | 42520517 ± 1420102 | 36506460 ± 2722413 |
| PC(35:1)+H | 774.6007 | 395.3 | $C_{43}H_{85}O_8NP$ | PC | (35:1) | 46644505 ± 1241858 | 44110089 ± 2605718 |
| PC(36:1e)+H | 774.6371 | 444.94 | $C_{44}H_{89}O_7NP$ | PC | (36:1e) | 23954908 ± 1318883 | 20255337 ± 1738660 |
| PC(36:4)+H | 782.5694 | 335.8 | $C_{44}H_{81}O_8NP$ | PC | (36:4) | 1044886321 ± 53040058 | 1041344788 ± 49769789 |
| PC(36:3)+H | 784.5851 | 347.92 | $C_{44}H_{83}O_8NP$ | PC | (36:3) | 22507124 ± 1136685 | 25113913 ± 1624553 |
| PC(36:2)+H | 786.6007 | 379.78 | $C_{44}H_{85}O_8NP$ | PC | (36:2) | 414842931 ± 25380771 | 386298512 ± 24150022 |
| PC(36:1)+H | 788.6164 | 418.98 | $C_{44}H_{87}O_8NP$ | PC | (36:1) | 1585506946 ± 52130483 | 1388590719 ± 78159047 |
| PC(36:0)+H | 790.632 | 458.34 | $C_{44}H_{89}O_8NP$ | PC | (36:0) | 13729129 ± 664620 | 11278022 ± 808573 |
| PC(37:4)+H | 796.5851 | 356.72 | $C_{45}H_{83}O_8NP$ | PC | (37:4) | 5036477 ± 318402 | 5163877 ± 310847 |
| PC(38:6)+H | 806.5694 | 325.1 | $C_{46}H_{81}O_8NP$ | PC | (16:0/22:6) | 728839570 ± 44568585 | 708650922 ± 84262687 |
| PC(38:5)+H | 808.5851 | 352.3 | $C_{46}H_{83}O_8NP$ | PC | (38:5) | 216968566 ± 13381802 | 204598735 ± 9959614 |

| | | | | | | | |
|---|---|---|---|---|---|---|---|
| PC(38:4)+H | 810.6007 | 384.86 | $C_{46}H_{85}O_8NP$ | PC | (38:4) | 684023657 ± 21587197 | 663037581 ± 24328338 |
| PC(38:3)+H | 812.6164 | 395.72 | $C_{46}H_{87}O_8NP$ | PC | (38:3) | 24989121 ± 1013201 | 23418016 ± 1113299 |
| PC(38:2)+H | 814.632 | 418.78 | $C_{46}H_{89}O_8NP$ | PC | (38:2) | 42225775 ± 1550380 | 35738330 ± 2619357 |
| PC(38:1)+H | 816.6477 | 461.9 | $C_{46}H_{91}O_8NP$ | PC | (38:1) | 62456377 ± 3339235 | 47281498 ± 4307697 |
| PC(40:7)+H | 832.5851 | 328.16 | $C_{48}H_{83}O_8NP$ | PC | (40:7) | 355461192 ± 10797655 | 325922338 ± 17770318 |
| PC(40:6)+H | 834.6007 | 366.18 | $C_{48}H_{85}O_8NP$ | PC | (40:6) | 255127030 ± 10697665 | 232849482 ± 12389867 |
| PC(40:5)+H | 836.6164 | 377.5 | $C_{48}H_{87}O_8NP$ | PC | (40:5) | 27630236 ± 536462 | 26569780 ± 1463193 |
| PC(40:4)+H | 838.632 | 402.94 | $C_{48}H_{89}O_8NP$ | PC | (40:4) | 53960384 ± 2131427 | 47504750 ± 2775447 |
| PC(40:2)+H | 842.6633 | 461.36 | $C_{48}H_{93}O_8NP$ | PC | (40:2) | 17655500 ± 1140947 | 12829565 ± 1345492 |
| PC(40:1)+H | 844.679 | 502.7 | $C_{48}H_{95}O_8NP$ | PC | (40:1) | 35394473 ± 2489364 | 26074984 ± 2441450 |
| PC(42:10)+H | 854.5694 | 288.94 | $C_{50}H_{81}O_8NP$ | PC | (42:10) | 4732431 ± 303338 | 4470863 ± 281836 |
| PC(42:8)+H | 858.6007 | 329.24 | $C_{50}H_{85}O_8NP$ | PC | (42:8) | 12192990 ± 352407 | 11197324 ± 634872 |
| PC(41:1)+H | 858.6946 | 524.56 | $C_{49}H_{97}O_8NP$ | PC | (41:1) | 6367321 ± 496274 | 4569069 ± 460865 |
| PC(42:7)+H | 860.6164 | 367.12 | $C_{50}H_{87}O_8NP$ | PC | (42:7) | 7249447 ± 302313 | 6141924 ± 371527 |
| PC(42:1)+H | 872.7103 | 539.62 | $C_{50}H_{99}O_8NP$ | PC | (42:1) | 22880015 ± 1745326 | 17619307 ± 1691091 |
| **Phosphatidylserine** | | | | | | | |

| | | | | | | | |
|---|---|---|---|---|---|---|---|
| PS(18:1/18:1)+H | 788.5436 | 339.7 | $C_{42}H_{79}O_{10}NP$ | PS | (18:1/18:1) | 30550480 ± 1191362 | 28036407 ± 1829513 |
| PS(18:0/18:1)+H | 790.5593 | 379.78 | $C_{42}H_{81}O_{10}NP$ | PS | (18:0/18:1) | 176364612 ± 6595837 | 157774854 ± 9710268 |
| PS(18:0/22:6)+H | 836.5436 | 325.3 | $C_{46}H_{79}O_{10}NP$ | PS | (18:0/22:6) | 375426076 ± 12434005 | 338561370 ± 20017339 |
| PS(18:0/22:5)+H | 838.5593 | 356.36 | $C_{46}H_{81}O_{10}NP$ | PS | (18:0/22:5) | 14585049 ± 752127 | 13074054 ± 628191 |
| PS(18:0/22:4)+H | 840.5749 | 372.04 | $C_{46}H_{83}O_{10}NP$ | PS | (18:0/22:4) | 43962289 ± 1111539 | 41412780 ± 1930131 |
| PS(22:4/22:6)+H | 884.5436 | 286.62 | $C_{50}H_{79}O_{10}NP$ | PS | (22:4/22:6) | 5942725 ± 276206 | 5433577 ± 350357 |
| **Phosphatidylinositol** | | | | | | | |
| PI(16:0/20:4)+$NH_4$ | 876.5597 | 292.92 | $C_{45}H_{83}O_{13}NP$ | PI | (16:0/20:4) | 8898107 ± 423108 | 8397330 ± 426502 |
| PI(18:1/20:4)+$NH_4$ | 902.5753 | 292.34 | $C_{47}H_{85}O_{13}NP$ | PI | (18:1/20:4) | 5297353 ± 270342 | 5002631 ± 278596 |
| PI(18:0/20:4)+$NH_4$ | 904.591 | 333.18 | $C_{47}H_{87}O_{13}NP$ | PI | (18:0/20:4) | 69548865 ± 2528047 | 63415266 ± 2948773 |
| **Negative ionisation mode** | | | | | | | |
| **Lysophosphatidylethanolamine** | | | | | | | |
| LPE(18:0)-H | 480.3096 | 146.28 | $C_{23}H_{47}O_7NP$ | LPE | (18:0) | 12494183 ± 340750 | 11719958 ± 827898 |
| LPE(22:6)-H | 524.2783 | 39.9 | $C_{27}H_{43}O_7NP$ | LPE | (22:6) | 5259986 ± 627249 | 4603514 ± 406515 |
| **Phosphatidylethanolamine** | | | | | | | |
| PE(16:0p/16:0)-H | 674.513 | 407.16 | $C_{37}H_{73}O_7NP$ | PE | (16:0p/16:0) | 6051977 ± 175405 | 5426539 ± 313374 |

| | | | | | | | |
|---|---|---|---|---|---|---|---|
| PE(16:0/18:1)-H | 716.5236 | 388.48 | $C_{39}H_{75}O_8NP$ | PE | (16:0/18:1) | 71714002 ± 939916 | 63409138 ± 4916605 |
| PE(16:0p/20:4)-H | 722.513 | 365.36 | $C_{41}H_{73}O_7NP$ | PE | (16:0p/20:4) | 110292352 ± 2987477 | 102374043 ± 9190520 |
| PE(16:0e/20:4)-H | 724.5287 | 374.5 | $C_{41}H_{75}O_7NP$ | PE | (16:0e/20:4) | 11655518 ± 644457 | 10540862 ± 1043294 |
| PE(18:1p/18:1)-H | 726.5443 | 413.9 | $C_{41}H_{77}O_7NP$ | PE | (18:1p/18:1) | 262311977 ± 6282014 | 221888578 ± 15989372 |
| PE(18:0e/18:1)-H | 730.5756 | 462.84 | $C_{41}H_{81}O_7NP$ | PE | (18:0e/18:1) | 30283301 ± 1190623 | 25295601 ± 1800753 |
| PE(16:0/20:4)-H | 738.5079 | 346.74 | $C_{41}H_{73}O_8NP$ | PE | (16:0/20:4) | 76434748 ± 1463504 | 72329402 ± 6337640 |
| PE(36:4)-H | 738.5079 | 648.66 | $C_{41}H_{73}O_8NP$ | PE | (36:4) | 7909748 ± 263045 | 7228706 ± 703078 |
| PE(18:1/18:2)-H | 740.5236 | 357.3 | $C_{41}H_{75}O_8NP$ | PE | (18:1/18:2) | 4996178 ± 226106 | 5160839 ± 509008 |
| PE(18:1/18:1)-H | 742.5392 | 390.42 | $C_{41}H_{77}O_8NP$ | PE | (18:1/18:1) | 78948705 ± 2011445 | 68797773 ± 5024460 |
| PE(18:0/18:1)-H | 744.5549 | 432.34 | $C_{41}H_{79}O_8NP$ | PE | (18:0/18:1) | 188602257 ± 3737102 | 167059069 ± 8136023 |
| PE(16:0p/22:6)-H | 746.513 | 352.54 | $C_{43}H_{73}O_7NP$ | PE | (16:0p/22:6) | 246605868 ± 4823285 | 223580365 ± 18516416 |
| PE(16:0e/22:6)-H | 748.5287 | 360.68 | $C_{43}H_{75}O_7NP$ | PE | (16:0e/22:6) | 115638837 ± 4557845 | 98423945 ± 9333402 |
| PE(18:0p/20:4)-H | 750.5443 | 404.2 | $C_{43}H_{77}O_7NP$ | PE | (18:0p/20:4) | 236589276 ± 4787893 | 230355851 ± 13192881 |
| PE(18:0p/22:6)-H | 774.5443 | 399.22 | $C_{45}H_{77}O_7NP$ | PE | (18:0p/22:6) | 398871100 ± 4386945 | 375275344 ± 21898034 |
| PE(18:0p/22:5)-H | 776.56 | 427.14 | $C_{45}H_{79}O_7NP$ | PE | (18:0p/22:5) | 17622931 ± 298452 | 15081144 ± 802522 |
| PE(18:2/22:6)-H | 786.5079 | 305.98 | $C_{45}H_{73}O_8NP$ | PE | (18:2/22:6) | 6504750 ± 166953 | 7173665 ± 668375 |

| | | | | | | | |
|---|---|---|---|---|---|---|---|
| PE(18:0/22:4)-H | 794.5705 | 421.2 | $C_{45}H_{81}O_8NP$ | PE | (18:0/22:4) | 94628112 ± 1727373 | 89680729 ± 5515864 |
| PE(20:0p/22:6)-H | 802.5756 | 441.24 | $C_{47}H_{81}O_7NP$ | PE | (20:0p/22:6) | 6115589 ± 68271 | 6084246 ± 315391 |
| PE(24:4/18:0)-H | 822.6018 | 454.5 | $C_{47}H_{85}O_8NP$ | PE | (24:4/18:0) | 5908148 ± 167841 | 5458527 ± 318142 |
| **Sphingomyelins** | | | | | | | |
| SM(d18:1/16:0)+HCOO | 747.5658 | 322.54 | $C_{40}H_{80}O_8N_2P$ | SM | (d18:1/16:0) | 4065172 ± 148097 | 3640179 ± 359766 |
| SM(d40:1)+HCOO | 831.6597 | 459.7 | $C_{46}H_{92}O_8N_2P$ | SM | (d40:1) | 7962239 ± 259331 | 6836804 ± 452914 |
| SM(d22:1/20:1)+HCOO | 857.6753 | 453.9 | $C_{48}H_{94}O_8N_2P$ | SM | (d22:1/20:1) | 36205637 ± 1083919 | 30604632 ± 2069913 |
| SM(d20:0/16:0)+HCOO | 777.6127 | 387.04 | $C_{42}H_{86}O_8N_2P$ | SM | (d20:0/16:0) | 4408764 ± 207855 | 3661766 ± 324815 |
| **Phosphatidylglycerol** | | | | | | | |
| PG(16:0/18:1)-H | 747.5182 | 338.68 | $C_{40}H_{76}O_{10}P$ | PG | (16:0/18:1) | 14084177 ± 247181 | 12263540 ± 855537 |
| PG(16:0/20:4)-H | 769.5025 | 303.64 | $C_{42}H_{74}O_{10}P$ | PG | (16:0/20:4) | 5698291 ± 183472 | 5835813 ± 496346 |
| PG(18:0/20:4)-H | 797.5338 | 342.54 | $C_{44}H_{78}O_{10}P$ | PG | (18:0/20:4) | 5135325 ± 184218 | 5000016 ± 398882 |
| PG(22:6/22:6)-H | 865.5025 | 240 | $C_{50}H_{74}O_{10}P$ | PG | (22:6/22:6) | 6903407 ± 189824 | 6730357 ± 587273 |
| **Phosphatidylserine** | | | | | | | |
| PS(16:0/18:1)-H | 760.5134 | 335.86 | $C_{40}H_{75}O_{10}NP$ | PS | (16:0/18:1) | 15280841 ± 417463 | 13030588 ± 1218442 |
| PS(18:1/18:1)-H | 786.5291 | 339.52 | $C_{42}H_{77}O_{10}NP$ | PS | (18:1/18:1) | 56504806 ± 1816231 | 48170540 ± 4657692 |

| | | | | | | | |
|---|---|---|---|---|---|---|---|
| PS(18:0/18:1)-H | 788.5447 | 378.52 | $C_{42}H_{79}O_{10}NP$ | PS | (18:0/18:1) | 179935107 ± 4777208 | 146475633 ± 14424381 |
| PS(16:0/22:6)-H | 806.4978 | 289.64 | $C_{44}H_{73}O_{10}NP$ | PS | (16:0/22:6) | 12223520 ± 1267910 | 11634111 ± 1510563 |
| PS(18:0/20:4)-H | 810.5291 | 341.86 | $C_{44}H_{77}O_{10}NP$ | PS | (18:0/20:4) | 70437615 ± 1779938 | 61959944 ± 5499022 |
| PS(18:0/20:1)-H | 816.576 | 416.52 | $C_{44}H_{83}O_{10}NP$ | PS | (18:0/20:1) | 20056213 ± 1772407 | 16461965 ± 1616123 |
| PS(39:0)-H | 832.6073 | 416.14 | $C_{45}H_{87}O_{10}NP$ | PS | (39:0) | 390765053 ± 5688384 | 345313606 ± 24611362 |
| PS(18:0/22:4)-H | 838.5604 | 368.92 | $C_{46}H_{81}O_{10}NP$ | PS | (18:0/22:4) | 56403819 ± 1940755 | 49839291 ± 4875249 |
| PS(41:5)-H | 850.5604 | 322.78 | $C_{47}H_{81}O_{10}NP$ | PS | (41:5) | 114752816 ± 1422025 | 101645443 ± 9385382 |
| PS(43:7)-H | 874.5604 | 298.64 | $C_{49}H_{81}O_{10}NP$ | PS | (43:7) | 4076522 ± 124030 | 4097940 ± 403580 |
| PS(22:6/22:6)-H | 878.4978 | 250.3 | $C_{50}H_{73}O_{10}NP$ | PS | (22:6/22:6) | 8076274 ± 183712 | 7356860 ± 667546 |
| PS(24:4/22:6)-H | 910.5604 | 319.4 | $C_{52}H_{81}O_{10}NP$ | PS | (24:4/22:6) | 5874390 ± 413455 | 4849389 ± 459667 |
| **Phosphatidylcholine** | | | | | | | |
| PC(16:0p/16:0)+HCOO | 762.5654 | 393.48 | $C_{41}H_{81}O_{9}NP$ | PC | (16:0p/16:0) | 5187291 ± 223598 | 4226922 ± 331095 |
| PC(16:0e/16:0)+HCOO | 764.5811 | 399.68 | $C_{41}H_{83}O_{9}NP$ | PC | (16:0e/16:0) | 6041154 ± 164798 | 5466009 ± 372591 |
| PC(16:0e/18:1)+HCOO | 790.5967 | 397.34 | $C_{43}H_{85}O_{9}NP$ | PC | (16:0e/18:1) | 57151889 ± 1035177 | 48568590 ± 3669999 |
| PC(16:0/18:2)+HCOO | 802.5604 | 341.62 | $C_{43}H_{81}O_{10}NP$ | PC | (16:0/18:2) | 39072481 ± 888473 | 41522110 ± 4006069 |
| PC(16:0/18:1)+HCOO | 804.576 | 375.38 | $C_{43}H_{83}O_{10}NP$ | PC | (16:0/18:1) | 638646014 ± 10246996 | 575723044 ± 52073044 |

| | | | | | | | |
|---|---|---|---|---|---|---|---|
| PC(17:0/18:1)+HCOO | 818.5917 | 397.02 | $C_{44}H_{85}O_{10}NP$ | PC | (17:0/18:1) | 8980789 ± 96651 | 8347490 ± 583352 |
| PC(18:0e/18:1)+HCOO | 818.628 | 446.74 | $C_{45}H_{89}O_{9}NP$ | PC | (18:0e/18:1) | 14698371 ± 480618 | 11817529 ± 967529 |
| PC(18:1/22:0)+HCOO | 888.6699 | 503.1 | $C_{49}H_{95}O_{10}NP$ | PC | (18:1/22:0) | 17114970 ± 574342 | 13970786 ± 884417 |
| **Phosphatidylinositol** | | | | | | | |
| PI(18:1/18:1)-H | 861.5499 | 328.02 | $C_{45}H_{82}O_{13}P$ | PI | (18:1/18:1) | 4748030 ± 298152 | 3372502 ± 402950 |
| PI(18:1/20:4)-H | 883.5342 | 298.78 | $C_{47}H_{80}O_{13}P$ | PI | (18:1/20:4) | 51384029 ± 1561335 | 48346716 ± 4255545 |
| PI(18:0/20:4)-H | 885.5499 | 327.28 | $C_{47}H_{82}O_{13}P$ | PI | (18:0/20:4) | 540882579 ± 15367450 | 473655304 ± 53326862 |

*m*/*z* denotes mass-to-charge ratios of the features used for identification; RT denotes retention time in seconds.

**Table S4.** Lipid species that were significantly different in the brain of rats fed human milk or infant formula ($n$ = 12) based on percentage contribution to respective class.

| **Lipid species** | **Human milk vs Infant formula** | **Lipid species** | **Human milk vs Infant formula** |
|---|---|---|---|
| **Positive ionization mode** | | **Negative ionization mode** | |
| CerG1(d18:0/24:0)+H | Human milk > Infant formula | PC(16:0p/16:0)+HCOO | Human milk > Infant formula |
| CerG1(d18:0/24:1)+H | Human milk > Infant formula | PC(18:0e/18:1)+HCOO | Human milk > Infant formula |
| CerG1(d18:1/22:1)+H | Human milk > Infant formula | PC(18:1/22:0)+HCOO | Human milk > Infant formula |
| CerG1(d18:1/24:2)+H | Human milk > Infant formula | PE(18:0/18:1)-H | Human milk > Infant formula |
| CerG1(d42:0+O)+H | Human milk > Infant formula | PE(18:0p/22:5)-H | Human milk > Infant formula |
| PC(34:1e)+H | Human milk > Infant formula | PG(16:0/18:1)-H | Human milk > Infant formula |
| PC(36:0)+H | Human milk > Infant formula | PI(18:1/18:1)-H | Human milk > Infant formula |
| PC(36:1)+H | Human milk > Infant formula | PS(18:0/18:1)-H | Human milk > Infant formula |
| PC(38:1)+H | Human milk > Infant formula | PC(16:0/18:2)+HCOO | Human milk < Infant formula |
| PC(40:1)+H | Human milk > Infant formula | PE(16:0/20:4)-H | Human milk < Infant formula |
| PC(40:2)+H | Human milk > Infant formula | PE(18:0p/20:4)-H | Human milk < Infant formula |
| PC(40:4)+H | Human milk > Infant formula | PE(18:1/18:2)-H | Human milk < Infant formula |
| PC(41:1)+H | Human milk > Infant formula | PE(18:2/22:6)-H | Human milk < Infant formula |
| PC(42:1)+H | Human milk > Infant formula | PG(16:0/20:4)-H | Human milk < Infant formula |
| PC(42:7)+H | Human milk > Infant formula | PS(43:7)-H | Human milk < Infant formula |
| PE(18:0/18:1)+H | Human milk > Infant formula | | |
| PE(18:0/20:1)+H | Human milk > Infant formula | | |
| PE(18:0e/18:1)+H | Human milk > Infant formula | | |
| PE(18:0p/16:0)+H | Human milk > Infant formula | | |
| PE(18:0p/18:1)+H | Human milk > Infant formula | | |
| PE(18:1p/20:1)+H | Human milk > Infant formula | | |
| SM(d40:2)+H | Human milk > Infant formula | | |
| Cer(d18:2/18:0)+H | Human milk < Infant formula | | |
| PC(34:2)+H | Human milk < Infant formula | | |
| PC(36:3)+H | Human milk < Infant formula | | |
| PE(16:0/20:4)+H | Human milk < Infant formula | | |

Significance tested by Fisher's LSD ($\alpha$ = 0.05).

**Table S5.** Nutritional composition analysis of human milk and infant formula samples.

| Test/Reference | Unit of measurement | Human milk | Infant formula |
|---|---|---|---|
| Total solids ISO 6731/IDF 21:2010 | g/100g | 11.2 | 12.9 |
| Fat ISO 7208/IDF 22:2008 | g/100g | 1.88 | 3.67 |
| Protein ISO 8968-2/IDF 20-2:2001 | g/100g | 1.10 | 1.46 |
| Carbohydrate 1.2.8. FSANZ calculation | g/100g | 8.10 | 7.40 |
| Energy 1.2.8. FSANZ calculation | kJ/100g | 225.00 | 287.00 |

**Table S6.** List of lipids identified by LipidSearch™ software.

| Group | Lipid name |
|---|---|
| **P-Choline** | Lysophosphatidylcholine |
| | Platelet-activating factor |
| | Phosphatidylcholine |
| **P-Ethanol Amine** | Lysophosphatidylethanolamine |
| | Lysodimethylphosphatidylethanolamine |
| | Phosphatidylethanolamine |
| | Dimethylphosphatidylethanolamine |
| **P-Serine** | Lysophosphatidylserine |
| | Phosphatidylserine |
| **P-Glycerol** | Lysophosphatidylglycerol |

| | |
|---|---|
| | Phosphatidylglycerol |
| **P-Inositol** | Lysophosphatidylinositol |
| | Phosphatidylinositol |
| | Phosphatidylinositol |
| | Phosphatidylinositol |
| | Phosphatidylinositol |
| **P-Ethanol** | Lysophosphatidylethanol |
| | Phosphatidylethanol |
| **P-Acid** | Lysophosphatidic acid |
| | Phosphatidic acid |
| | Cyclic phosphatidic acid |
| **P-Methanol** | Lysophosphatidylmethanol |
| | Phosphatidylmethanol |
| **Sphingolipids** | Sphingomyelin |
| | Lysosphingomyelin |
| | Sphingomyelin(phytosphingosine) |
| **Neutral glycerolipid** | Monoglyceride |
| | Diglyceride |
| | Triglyceride |
| **Fatty Acid** | Fatty acid |
| | (O-acyl)-1-hydroxy fatty acid |

| | |
|---|---|
| **Cardiolipin** | Cardiolipin |
| **Sphingoid base** | Sphingoshine |
| | Sphingoshine phosphate |
| **Neutral Glycosphingolipids** | Glucosylsphingoshine |
| | Simple glc series |
| | Simple glc series |
| | Simple glc series |
| | Simple glc series |
| | Simple glc series |
| | Simple glc series |
| **Glycosphingolipids** | Ceramides |
| | Ceramides phosphate |
| | Gangliosides |
| | Gangliosides |
| | Gangliosides |
| | Gangliosides |
| | Gangliosides |
| | Gangliosides |
| | Gangliosides |
| | Gangliosides |
| | Gangliosides |

| | |
|---|---|
| | Gangliosides |
| | Gangliosides |
| | Gangliosides |
| | Gangliosides |
| | Gangliosides |
| **Steroid** | Cholesteryl ester |
| | Zymosteryl ester |
| | Stigmasteryl ester |
| | Sitosteryl ester |
| | Deuterated cholesteryl ester |
| **Coenzyme** | Coenzyme |
| **Glycoglycerolipid** | Monogalactosylmonoacylglycerol |
| | Monogalactosyldiacylglycerol |
| | Digalactosylmonoacylglycerol |
| | Digalactosyldiacylglycerol |
| | Sulfoquinovosylmonoacylglycerol |
| | Sulfoquinovosyldiacylglycerol |
| **Neutral glycerolipid (deuterated)** | Deuterated diglyceride |
| | Deuterated triglyceride |
